# Selective advantage of mutant stem cells in human clonal hematopoiesis is associated with attenuated response to inflammation and aging

**DOI:** 10.1016/j.stem.2024.05.010

**Published:** 2024-08-01

**Authors:** Niels Asger Jakobsen, Sven Turkalj, Andy G.X. Zeng, Bilyana Stoilova, Marlen Metzner, Susann Rahmig, Murtaza S. Nagree, Sayyam Shah, Rachel Moore, Batchimeg Usukhbayar, Mirian Angulo Salazar, Grigore-Aristide Gafencu, Alison Kennedy, Simon Newman, Benjamin J.L. Kendrick, Adrian H. Taylor, Rasheed Afinowi-Luitz, Roger Gundle, Bridget Watkins, Kim Wheway, Debra Beazley, Alex Murison, Alicia G. Aguilar-Navarro, Eugenia Flores-Figueroa, Stephanie G. Dakin, Andrew J. Carr, Claus Nerlov, John E. Dick, Stephanie Z. Xie, Paresh Vyas

**Affiliations:** 1 MRC Molecular Haematology Unit, MRC Weatherall Institute of Molecular Medicine, University of Oxford, Oxford, UK; 2 Oxford Centre for Haematology, NIHR Oxford Biomedical Research Centre, Oxford, UK; 3 Princess Margaret Cancer Centre, University Health Network, Toronto, ON, Canada; 4 Department of Molecular Genetics, University of Toronto, Toronto, ON, Canada; 5 Wellcome - MRC Cambridge Stem Cell Institute, University of Cambridge, Cambridge, UK; 6 Nuffield Department of Orthopaedics, Rheumatology and Musculoskeletal Sciences, Botnar Research Centre, University of Oxford, Oxford, UK; 7 Nuffield Orthopaedic Centre, Oxford University Hospitals NHS Foundation Trust, Oxford, UK; 8 Unidad de Investigación Médica en Enfermedades Oncológicas, Centro Médico Nacional Siglo XXI, Instituto Mexicano del Seguro Social, Mexico City, Mexico; 9 Department of Haematology, Oxford University Hospitals NHS Foundation Trust, Oxford, UK

**Keywords:** clonal hematopoiesis, hematopoietic stem cells, DNMT3A, TET2, aging, single-cell genomics, single-cell RNA-seq, clonal competition, somatic mosaicism

## Abstract

Clonal hematopoiesis (CH) arises when hematopoietic stem cells (HSCs) acquire mutations, most frequently in the *DNMT3A* and *TET2* genes, conferring a competitive advantage through mechanisms that remain unclear. To gain insight into how CH mutations enable gradual clonal expansion, we used single-cell multi-omics with high-fidelity genotyping on human CH bone marrow (BM) samples. Most of the selective advantage of mutant cells occurs within HSCs. *DNMT3A*- and *TET2*-mutant clones expand further in early progenitors, while *TET2* mutations accelerate myeloid maturation in a dose-dependent manner. Unexpectedly, both mutant and non-mutant HSCs from CH samples are enriched for inflammatory and aging transcriptomic signatures, compared with HSCs from non-CH samples, revealing a non-cell-autonomous effect. However, *DNMT3A*- and *TET2*-mutant HSCs have an attenuated inflammatory response relative to wild-type HSCs within the same sample. Our data support a model whereby CH clones are gradually selected because they are resistant to the deleterious impact of inflammation and aging.

## Highlights

• High-fidelity single-cell genotyping and transcriptomes of human clonal hematopoiesis
• Most clonal expansion occurs in HSCs; *TET2*^MUT^ clones also have a myeloid bias
• HSCs from CH samples show increased inflammation and aging-related gene expression
• These deleterious effects are attenuated in *DNMT3A*^MUT^ and *TET2*^MUT^ HSCs

## Graphical abstract

**Figure:**
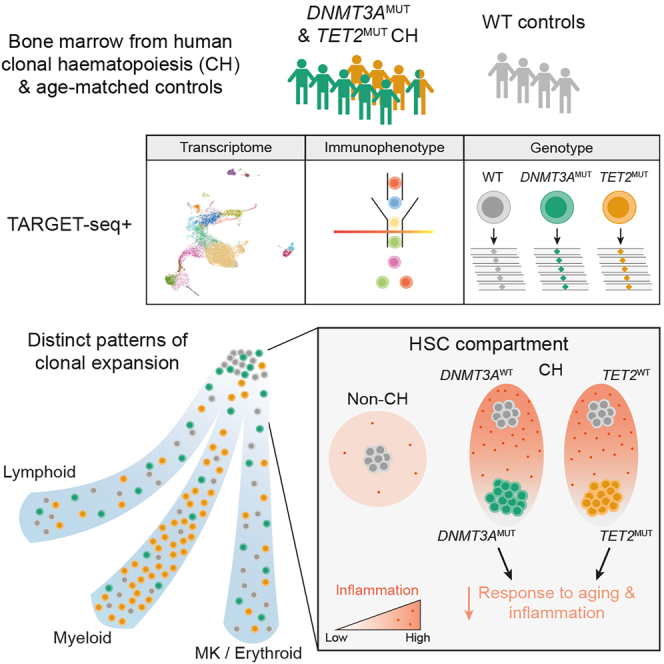

## Teaser

Vyas and colleagues show *DNMT3A* and *TET2* mutation-specific patterns of clonal expansion in human clonal hematopoiesis (CH). Hematopoietic stem cells (HSCs) in CH bone marrow have heightened transcriptional responses to inflammation. However, these responses are attenuated in CH mutant HSCs, and this could contribute to their clonal advantage.

## Introduction

Somatic mutations acquired in long-lived stem cells may provide a substrate for clonal selection, resulting in somatic mosaicism.^1^^,^^2^ With aging, somatic mosaicism becomes common in phenotypically normal human tissues,^3^^,^^4^^,^^5^^,^^6^^,^^7^^,^^8^^,^^9^^,^^10^^,^^11^^,^^12^ including the blood, where it is termed clonal hematopoiesis (CH).^13^^,^^14^^,^^15^^,^^16^ CH is associated with an elevated risk of myeloid malignancy, cardiovascular disease, and other adverse outcomes,^14^^,^^15^^,^^16^^,^^17^^,^^18^ and these risks increase with clone size.^17^^,^^18^^,^^19^^,^^20^^,^^21^^,^^22^ Though multiple mechanisms may contribute to clonal expansion,^23^ the biological principles remain unclear.

Interestingly, ∼70% of CH cases are associated with mutations in *DNMT3A* and *TET2*.^14^^,^^15^^,^^16^^,^^17^^,^^19^^,^^24^ DNMT3A, a *de novo* DNA methyltransferase, catalyzes the conversion of cytosine to 5-methylcytosine (5mC), usually in CpG dinucleotides.^25^ TET2 is a dioxygenase that catalyzes the conversion of 5mC to 5-hydroxymethylcytosine (5hmC) and other oxidized derivatives.^26^^,^^27^ This reaction is the first step in DNA demethylation, although 5hmC can also play a regulatory role.^28^^,^^29^ Both proteins have been associated with additional functions beyond DNA modification.^30^^,^^31^^,^^32^^,^^33^^,^^34^^,^^35^^,^^36^^,^^37^ In CH, *DNMT3A* mutations are predominantly heterozygous, scattered throughout the three functional domains, and predicted to cause loss of function (LoF). By contrast, ∼60% of *DNMT3A* mutations in acute myeloid leukemia affect the R882 residue in the methyltransferase domain. *TET2* mutations are missense or truncating variants distributed across the coding region and are predicted to inhibit or abolish the enzyme’s catalytic activity.

In mice, *Dnmt3a*^*−/−*^ hematopoietic stem cells (HSCs) outcompete wild-type (WT) HSCs in competitive transplants, which has been attributed to increased RNA expression of multipotency and self-renewal genes.^38^ Similarly, *Tet2*^*−/−*^ and *Tet2*^*+/−*^ HSCs have a competitive advantage in transplantation assays.^39^^,^^40^^,^^41^^,^^42^^,^^43^ HSC differentiation is also altered, where deletion of *Dnmt3a* increases megakaryocyte-erythroid (MEP) and decreases myelomonocytic cells,^44^ whereas *Tet2* loss confers a myeloid bias^41^ with reduction of MEP and lymphoid progenitors.^44^ Notably, infection and inflammation increase clonal advantage of *Dnmt3a*^*−/−*^, *Tet2*^*−/−*^, and *Tet2*^*+/−*^ HSCs, which is abrogated by deletion of several cytokine receptors.^45^^,^^46^^,^^47^^,^^48^^,^^49^^,^^50^

However, murine studies may not capture the complexities of human CH, which arises when a single cell acquires a mutation conferring selective advantage, leading to gradual clonal expansion over time. Indeed, recent studies using population genetic modeling, single-cell phylogenetic analysis, and longitudinal sampling have estimated that *DNMT3A* and *TET2* mutant CH clones expand by about 5%–20% per year and are acquired decades before reaching a substantial clone size.^51^^,^^52^^,^^53^ By contrast, mice are kept in controlled environments, have short lifespans, and murine studies often assay hematopoiesis after transplantation or introduction of a mutant allele in all blood cells.

In humans, detailed single-cell analyses have been performed in *DNMT3A*^R882^-mutated CH from patients treated for myeloma,^54^ and in cord blood hematopoietic stem and progenitor cells (HSPCs) where *TET2* was experimentally deleted.^55^^,^^56^ In both settings, differentiation defects were observed, with engineered *TET2*^*−/−*^ cells having a competitive advantage *in vivo* in immunodeficient mice. However, it remains unclear how *DNMT3A* and *TET2* mutations confer a gradual clonal advantage in age-associated CH. To address this question, we implemented an optimized single-cell multi-omics method combining high-fidelity genotyping with transcriptional profiling to separately analyze mutant and non-mutant HSPCs in humans with CH.

## Results

### BM sampling from individuals with age-related CH without prior hematological malignancy

**Figure 1:**
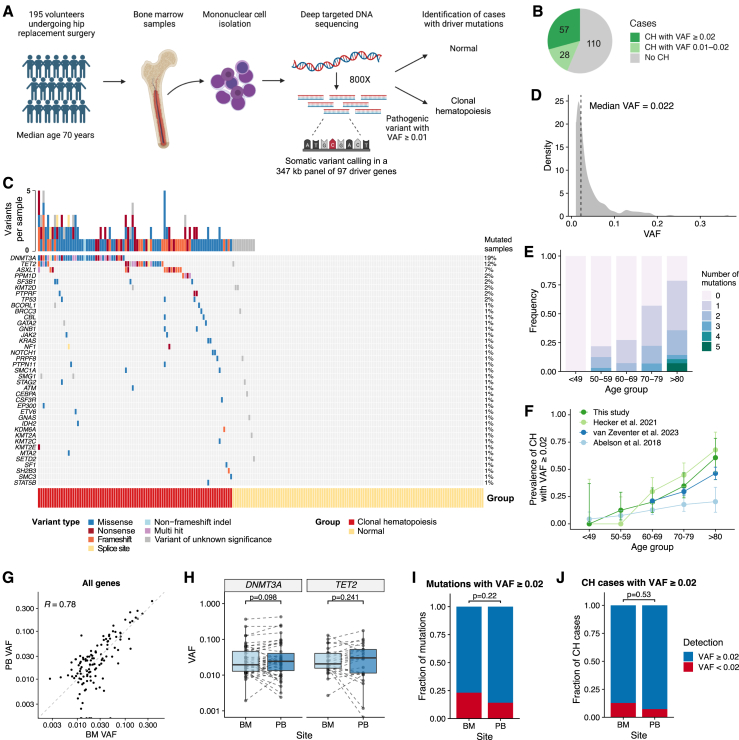
Identification of age-related clonal hematopoiesis in individuals undergoing hip replacement surgery (A) Experimental design for identifying individuals with clonal hematopoiesis (CH). (B) Fraction of samples in the cohort with driver mutation(s) at 0.01–0.02 or ≥0.02 VAF. (C) Landscape of somatic variants observed in the cohort. Each row represents a gene, and each column represents a study participant. Top bar plot indicates the number of mutations per sample. Variants are classified as pathogenic or variants of unknown significance (VUSs) (STAR Methods). Samples with ≥1 pathogenic driver mutation were categorized as having CH. (D) Distribution of VAFs in all mutations observed across the cohort. (E) Frequency of mutations detected per individual by age group. (F) Prevalence of CH with driver mutation(s) ≥0.02 VAF by age. BM sequencing data from this study are compared with another hip replacement cohort (Hecker et al.^57^; *n* = 109 BM and *n* = 91 PB samples; green colors) and with two studies of the general population^19^^,^^58^ (blue colors). Error bars represent 95% confidence intervals (CIs). (G) Comparison of VAFs for 128 mutations in paired BM and PB samples. Mutations detected with VAF ≥0.01 in either sample type were included. The dashed line shows the line of equality where BM VAF is equal to PB VAF. *R* indicates the Pearson correlation coefficient. (H) Pairwise comparison of VAFs for mutations in *DNMT3A* (*n* = 35) and *TET2* (*n* = 19). Significance calculated by Wilcoxon signed-rank test. (I and J) Proportion of mutations (I) or CH cases (J) detected with ≥0.02 VAF in BM or PB DNA (*n* = 83 cases with paired BM and PB data). Significance calculated by Fisher’s test.

To obtain bone marrow (BM) samples from individuals with CH without the confounding effects of co-existing malignancy, we collected samples from 195 individuals with normal blood counts undergoing elective total hip replacement surgery for osteoarthritis. To study steady-state CH, we excluded subjects with prior or current hematological cancer, inflammatory arthritis, or systemic steroid use (Table S1). All samples underwent targeted re-sequencing of BM mononuclear cell (MNC) DNA, using a 97-gene panel with a mean sequencing depth of 822× (Figures 1A and S1A; Table S2). 57 individuals (29.2%) had CH with somatic driver mutation(s) at ≥0.02 variant allele frequency (VAF), and an additional 28 individuals (14.3%) had mutation(s) at 0.01–0.02 VAF (Figures 1B and 1C; Table S2). Consistent with prior studies, 69% of CH cases had mutations in *DNMT3A* and *TET2* (Figures 1C and S1B). The median VAF detected was 0.022 (Figure 1D). Most cases had a single mutation, but the frequency of secondary mutations increased with age (Figure 1E).

CH prevalence increased with age (Figures 1F and S1C). Comparison with previous studies^19^^,^^57^^,^^58^ showed the prevalence in our cohort was similar to a study of peripheral blood (PB) DNA from 3,359 individuals in the general population^58^ and to another hip replacement surgery cohort^57^ (Figures 1F and S1C).

Most studies of CH to date have performed sequencing on PB DNA. To determine whether the sensitivity for CH detection differs between BM and PB, we compared mutation detection in paired PB granulocytic and BM MNC DNA on 72 samples with CH and 27 samples without CH. PB sequencing identified 14 additional mutations not called in BM sequencing. Although VAFs for some mutations differed considerably between BM and PB, there was no significant difference overall (Figure 1G; *p* = 0.11; Wilcoxon signed-rank test) or specifically for *DNMT3A* and *TET2* mutations (Figure 1H). There was no significant difference in the number mutations with ≥0.02 VAF or individuals with CH detected between BM and PB (Figures 1I and 1J). Our analysis suggests somatic mutations are detected with comparable sensitivity in PB and BM and that the frequency of CH is similar between patients undergoing hip replacement surgery and the general population.

### HSPC differentiation landscape in *DNMT3A*- and *TET2*-mutant CH

As mutant cells in CH occur at low frequency, a high-fidelity method for distinguishing mutant and WT cells within the same sample is needed to accurately study the consequences of CH mutations. To address this, we optimized TARGET-seq,^59^ which combines high-fidelity single-cell genotyping with transcriptome sequencing on flow cytometry index-sorted cells. Our new method, TARGET-seq+, incorporates elements of Smart-seq3 chemistry^60^ to increase transcript detection sensitivity. We compared TARGET-seq and TARGET-seq+ on JURKAT cells and primary human lineage^−^ (Lin^−^) CD34^+^ HSPCs (Figure S1D). Sequencing metrics were comparable (Figure S1E), but TARGET-seq+ yielded a higher proportion of cells passing quality filters (Figure S1F) and increased the number of genes detected per cell by 13.5% in JURKAT cells and by 19.0% in HSPCs (Figures S1G and S1H). Increased detection was observed in both frequently and lowly expressed genes (Figure S1I). Consistent with better transcript capture, cell-to-cell correlations of transcript levels improved with TARGET-seq+ (Figure S1J).

**Figure 2:**
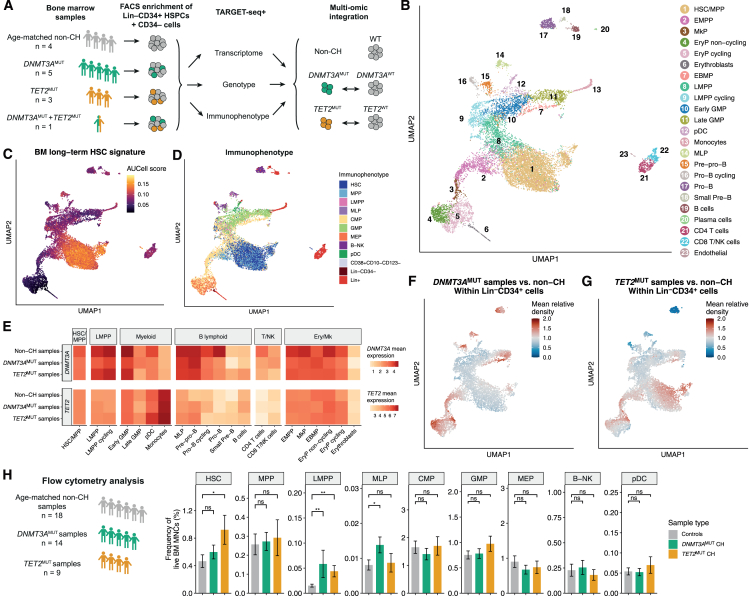
Hematopoietic differentiation trajectory in *DNMT3A* and *TET2*-mutant clonal hematopoiesis (A) Experimental design for TARGET-seq+ analysis of BM samples from donors with CH and age-matched samples without CH. See Figure S1. (B) Uniform manifold approximation and projection (UMAP) of integrated single-cell transcriptome data (*n* = 13,247 cells from 13 donors) colored by cluster annotation. See Figure S2. (C) UMAP with AUCell enrichment scores for the BM long-term HSC signature.^61^ (D) UMAP colored by cell immunophenotype determined from flow cytometry indexing. (E) Heatmap of mean log_2_(normalized counts) for *DNMT3A* and *TET2* in control, *DNMT3A*-, and *TET2*-mutant samples across hematopoietic cell types. (F and G) UMAPs colored by the mean density of Lin^−^CD34^+^ cells in *DNMT3A*-mutant (F) and *TET2*-mutant (G) CH samples relative to non-CH samples. Relative density >1 indicates that the probability of observing a given cell is greater in CH samples than in non-CH samples. (H) Flow cytometry analysis on BM samples from non-CH samples and CH samples with either *DNMT3A* or *TET2* mutations present in the largest clone. Data are represented as mean ± SEM. *p* values calculated by Wilcoxon rank-sum test with Holm-Bonferroni multiple testing correction. ^∗^*p* < 0.05, ^∗∗^*p* < 0.01. GMP, granulocyte-monocyte progenitor; pDC, plasmacytoid dendritic cell progenitor; MkP, megakaryocytic progenitor; EryP, erythroid progenitor; EBMP, eosinophil/basophil/mast cell progenitor; MLP, multi-lymphoid progenitor; B-NK, B/NK cell progenitor.

We applied TARGET-seq+ to 9 CH samples and 4 age-matched controls without known driver mutations (non-CH samples) (Figure 2A; Table S3). We focused on *DNMT3A*-mutant (*DNMT3A*^MUT^) and *TET2*-mutant (*TET2*^MUT^) CH by selecting 5 cases with heterozygous *DNMT3A* LoF mutations, 3 with *TET2* LoF mutations, and one case with mutations in both *DNMT3A* and *TET2*. VAFs in BM ranged from 0.061 to 0.366 (Table S3). In two CH cases, there were additional mutations in other genes at VAF < 0.02 (Table S3). We sorted a mean of 1,071 cells/sample (range 348–1,824), composed of purified Lin^−^CD34^+^ HSPCs, further enriched for primitive Lin^−^CD34^+^CD38^−^ HSCs and multipotent progenitors (MPPs), combined with CD34^−^ mature cells (Figure S1K). Approximately 40%–45% of cells across all samples were Lin^−^CD34^+^CD38^−^, a similar percentage were Lin^−^CD34^+^CD38^+^, and ∼10% were CD34^−^ (Figure S1L). 95.0% of sorted cells (13,247/13,939) were included for further analysis after quality filtering (Figure S1M). We detected ∼10^6^ RNA counts (Figure S1N) and a median of 6,484 genes per cell (Figure S1O). Importantly, these metrics were consistent across samples.

To generate a hematopoietic landscape based on single-cell RNA profiles, we integrated gene expression data across the 13 samples (Figure S2A). We annotated 23 cell clusters using published gene signatures^61^^,^^62^^,^^63^^,^^64^^,^^65^^,^^66^^,^^67^^,^^68^^,^^69^ and marker genes (Figures 2B, 2C, S2B, and S2C). Immunophenotypic HSPC populations, defined from flow cytometry index data, were often present in multiple transcriptionally defined populations (Figures 2D and S2D), consistent with transcriptional clusters providing a more granular view of hematopoietic cell states.^62^^,^^67^

Downstream of HSC/MPPs, a continuum of cell states was observed, with initial separation into lymphoid-primed MPPs (LMPPs) and erythroid/megakaryocytic-primed MPPs (EMPPs) (Figure 2B). A series of cell states with neutrophilic/monocytic and lymphoid potential extended downstream of LMPPs, whereas progressing from the EMPP were erythroid cells and megakaryocytes, consistent with prior data.^64^^,^^70^ *DNMT3A* and *TET2* were expressed in HSC/MPP, LMPP, and EMPP but decreased in late lymphoid and erythroid lineages (Figure 2E). Notably, *TET2* expression increased during myeloid maturation, in contrast to *DNMT3A*.

We first asked whether the size of HSPC compartments is perturbed in CH, given that their frequencies change with age^71^ and in myeloid disease.^72^^,^^73^^,^^74^^,^^75^ Comparing the frequency of cells across the transcriptional landscape between CH and non-CH samples (STAR Methods) showed moderate differences in various populations (Figures 2F and 2G), but these were not significant (at false discovery rate [FDR] < 0.2 using scCODA^76^; Figures S2E and S2F). Interestingly, conventional immunophenotyping on a larger sample set showed Lin^−^CD34^+^CD38^−^CD45RA^−^CD90^+^ HSCs and CD49f^+^ long-term HSCs (LT-HSCs) were 2- and 2.2-fold expanded, respectively, in *TET2*-mutant relative to non-CH samples (Figures 2H and S2G). Additionally, *DNMT3A*- and *TET2*-mutant CH samples showed a 3.9- and 2.9-fold increase in the rare Lin^−^CD34^+^CD38^−^CD45RA^+^ LMPP population, respectively (Figure 2H), which constitutes a minor fraction of the transcriptionally defined LMPP and multi-lymphoid progenitors (MLPs) (Figure S2D). Overall, the size of HSPC compartments is moderately perturbed in *DNMT3A*- and *TET2*-mutant CH.

### Distinct patterns of clonal expansion of *DNMT3A*- and *TET2*-mutant clones

Next, we integrated genotypes with transcriptional profiles to identify mutant and WT cells within the same sample. To accurately call genotypes while accounting for noise in PCR amplification and sequencing, we simultaneously genotyped cells from a WT control sample, which allowed us to determine the error rate at each locus (Figures S3A and S3B). Overall, *DNMT3A* and *TET2* loci were successfully amplified in 97.7% and 98.1% of cells, respectively, resulting in clonal assignment for 93.0% of CH cells (Figure S3C). Genotyping rates for *DNMT3A* and *TET2* were high across all samples (Figure S3C) and cell types (Figure S3D).

Loss of one allele during PCR amplification, termed allelic dropout (ADO), is common in single-cell genotyping and can compromise the accuracy of downstream analysis due to misassignment of heterozygous mutant cells as WT. We used two strategies to estimate ADO. In four loci, heterozygous germline single nucleotide polymorphisms (SNPs) showed ADO rates of 5.3%–13.8% (Figures S3E–S3H). For the remaining loci, where we inferred ADO rates from the frequency of cells that appeared homozygous mutant (i.e., where we assumed there was ADO of the WT allele; Figure S3I), these rates were 0%–13.5% (Figure S3J). The frequency of mutant cells detected by TARGET-seq+ correlated well with estimates from bulk analysis of Lin^−^CD34^+^ cells (*R* = 0.98), validating our single-cell genotyping (Figure S3K). A potential alternative way to assess genotyping accuracy would be to quantify expression of a gene specific for a certain genotype. It has been suggested that *TCL1A* is upregulated in *TET2*^MUT^ HSCs among individuals without the rs2887399 variant.^77^ Across *TET2*^MUT^ CH samples, 0.36%–2.5% of WT HSC/MPPs had >2 reads mapping the *TCL1A* gene (*TCL1A*^hi^), a higher rate compared with non-CH samples (Figure S3L). However, these *TCL1A*^hi^*TET2*^WT^ cells transcriptionally resembled other *TET2*^WT^ cells rather than *TET2*^MUT^ cells (Figures S3M–S3O). Therefore, in our dataset, *TCL1A*^hi^ status was not specific to *TET2*^MUT^ HSC/MPPs.

**Figure 3:**
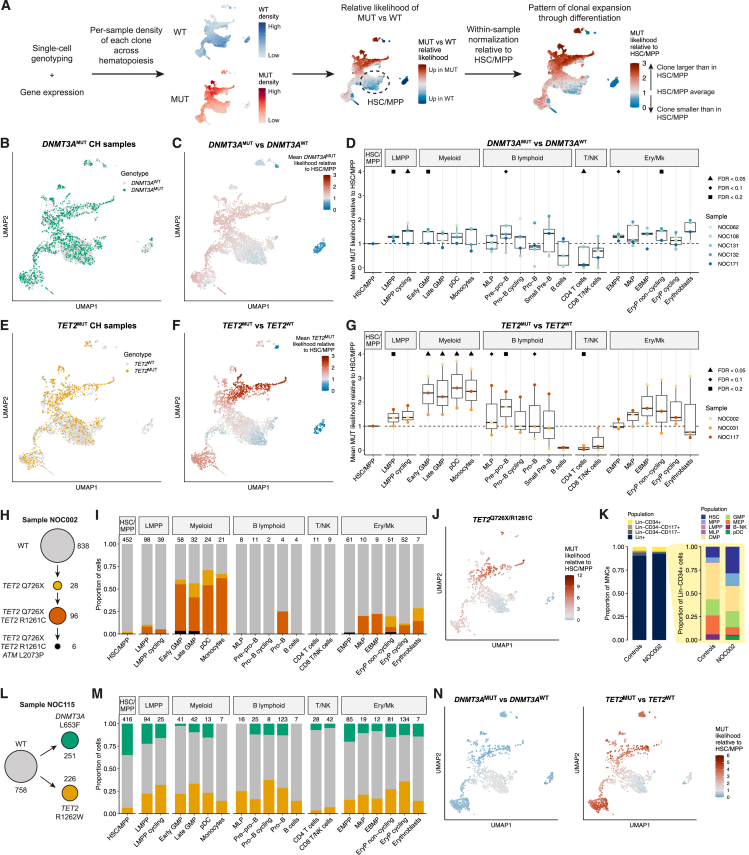
Distinct patterns of clonal expansion of *DNMT3A*- and *TET2*-mutant clones (A) Strategy for quantifying CH^MUT^ clonal expansion across hematopoietic differentiation. For each sample, MELD and scCODA were used to estimate the density of cells from each clone across the transcriptomic landscape (see Figures S3 and S4 and STAR Methods). A mutant relative likelihood >1 indicates that the probability of a cell being mutant is greater than in the HSC/MPP, whereas a relative likelihood <1 indicates that the probability is lower than in the HSC/MPP. (B) UMAP of cells from *DNMT3A*^MUT^ CH samples (*n* = 5 samples) colored by genotype. (C) UMAP of cells from *DNMT3A*^MUT^ CH samples colored by the mean likelihood of cells being *DNMT3A*^MUT^ relative to the average within HSC/MPP. The mean value across 5 samples is shown. (D) Mean *DNMT3A*^MUT^ clone likelihood in each cluster relative to the HSC/MPP, computed using MELD. Each dot represents a *DNMT3A*^MUT^ CH sample. Boxplots display the median and interquartile range. Symbols above indicate whether a significant difference in clone size relative to the HSC/MPP was detected using scCODA. (E) UMAP of cells from *TET2*^MUT^ CH samples (*n* = 3 samples) colored by genotype. (F) UMAP of cells from *TET2*^MUT^ CH samples colored by the mean likelihood of cells being *TET2*^MUT^ relative to the average within HSC/MPP. The mean value across 3 samples is shown. (G) Same as in (D), but for the *TET2*^MUT^ clone likelihoods across *TET2*^MUT^ CH samples. (H) Clonal structure for the NOC002 sample. Cell numbers in each clone are indicated. (I) Clonal composition within each cluster for sample NOC002. Each clone is colored as in (H). The number of cells analyzed in each cluster is shown above. (J) UMAP showing the likelihood of cells being in the double *TET2*^MUT^ clone (*TET2*^Q726X/R1261C^) relative to the average within HSC/MPP in sample NOC002. (K) Immunophenotypic BM compartment sizes comparing sample NOC002 with the median from 18 age-matched control samples. Left-hand bars: compartments as a proportion of total BM MNCs; right-hand bars: HSPC compartments within Lin^−^CD34^+^ cells. (L) Clonal structure for the NOC115 sample. The *DNMT3A* and *TET2* mutations were mutually exclusive in single-cell genotyping. Cell numbers in each clone are indicated. (M) As in (I) but for sample NOC115. Each clone is colored as in (L). (N) UMAPs showing the likelihood of cells being in the *DNMT3A*^MUT^ (left) and *TET2*^MUT^ (right) clones relative to the average within HSC/MPP in sample NOC115.

In all subjects, *DNMT3A* or *TET2* mutations were present in the founder clone, allowing us to determine the effect of mutations in a WT background (Figures S4A–S4H). To reveal how mutant clones expand or contract with differentiation, we projected genotypes onto the transcriptomic landscape and compared the density of mutant and WT cells (referred to as the mutant clone likelihood) to quantify how clone size changed relative to HSC/MPP (Figure 3A; STAR Methods).

*DNMT3A*^MUT^ cells were intermingled with *DNMT3A*^WT^ cells, both within individual samples (Figures S4A–S4E) and in the integrated dataset (Figure 3B), indicating that they shared a similar differentiation trajectory and that mutant cells did not create novel transcriptional states. In HSC/MPPs, the *DNMT3A*^MUT^ clone size was highly variable, ranging from 3.4% to 73.3% (Figures S4A–S4E). Changes in *DNMT3A*^MUT^ clone size through differentiation were modest (Figures 3C and 3D). Relative to HSC/MPPs, the mean mutant clone size was approximately 30% larger in early EMPP and LMPP populations (Figures 3C, 3D, and S4I). Clone sizes were largely maintained at later stages of differentiation, except in T cells, where clones were on average 50% smaller than in HSC/MPPs. Aside from this, there was no consistent lineage bias, but there was notable variability among individuals. While one sample showed almost complete absence of *DNMT3A*^MUT^ cells in lymphoid cells (Figure S4A), consistent with previous data from PB populations,^78^^,^^79^^,^^80^ another showed depletion of *DNMT3A*^MUT^ cells in myeloid progenitors (Figure S4D). Overall, *DNMT3A*^MUT^ clonal expansion occurred primarily in HSCs and early MPPs without consistent lineage bias.

*TET2*^MUT^ cells also intermingled with *TET2*^WT^ cells (Figure 3E). Clone size within HSC/MPPs was 1.1%–32.8% (Figures S4F–S4H). In contrast to *DNMT3A*^MUT^ clones, there was pronounced expansion of *TET2*^MUT^ clones downstream of HSC/MPPs during myelopoiesis (Figures 3F, 3G, and S4I). Indeed, *TET2*^MUT^ clones were on average 2.5- to 3-fold larger in granulocyte-monocyte progenitors (GMPs) compared with HSC/MPPs. In 2 of 3 samples, the mutant clone also expanded within erythroid progenitors (Figures S4G and S4H). There was heterogeneity in the contribution of *TET2*^MUT^ cells to lymphoid progenitors, where mutant cells were depleted in 2 cases (Figures S4F and S4H), but the *TET2*^MUT^ clone constituted 90% of B cell progenitors in another individual (Figure S4G). By contrast, *TET2*^MUT^ cells were almost absent from mature B and T cells in all samples, suggesting inability to complete lymphoid differentiation.

Next, to understand the differentiation potential of heterozygous versus homozygous *TET2*^MUT^ clones, we studied one sample where two *TET2* mutations were acquired sequentially in a linear clonal structure (Figure 3H). The single- and double-*TET2*^MUT^ clones each contributed to only 1.1% of HSC/MPPs. While the single-*TET2*^MUT^ clone expanded 3- to 4-fold in erythroid and myeloid progenitors, the double-*TET2*^MUT^ clone dramatically outcompeted the single mutant clone during myelopoiesis, contributing to >50% of GMPs, plasmacytoid dendritic cells (pDCs), and monocytes (Figures 3I and 3J), suggesting a dose-dependent effect of *TET2* LoF on myeloid clonal expansion. These findings were validated by droplet digital PCR (ddPCR) genotyping of immunophenotypic populations (Figure S4J). Interestingly, though 97.6% of HSC/MPPs were *TET2*^WT^, there was a 2.6-fold expansion of immunophenotypic HSC/MPPs in this sample relative to non-CH samples (Figure 3K). This raises the question of whether *TET2*^MUT^ CH might increase WT HSC/MPP cell numbers in a non-cell-autonomous manner.

Finally, we had the opportunity to study clonal competition between *DNMT3A*^MUT^ and *TET2*^MUT^ cells in an individual with co-existing, independent *DNMT3A*^MUT^ and *TET2*^MUT^ clones (Figure 3L). Interestingly, the *DNMT3A*^MUT^ clone was 5 times larger than the *TET2*^MUT^ clone within HSC/MPPs, but the *TET2*^MUT^ clone became 4 times larger than the *DNMT3A*^MUT^ clone within GMPs (Figures 3M and 3N). Notably, the *TET2*^MUT^ clone was also larger than the *DNMT3A*^MUT^ clone in erythroid and lymphoid progenitors (Figure 3M), observations once again validated by ddPCR (Figure S4K).

In summary, *DNMT3A*^MUT^ and *TET2*^MUT^ clones showed distinct patterns of clonal expansion across differentiation. The selective advantage of *DNMT3A*^MUT^ clones occurs mainly in HSCs and early MPPs, whereas *TET2*^MUT^ clones expand in HSCs and further through differentiation, especially in myelopoiesis.

### Transcriptional basis for dysregulated myeloid differentiation of *TET2*-mutant clones

**Figure 4:**
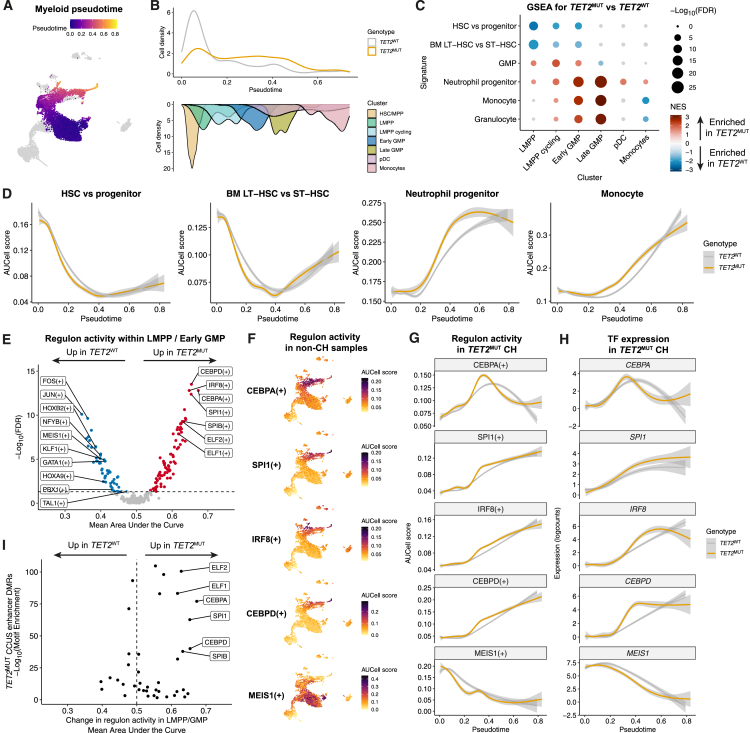
*TET2*-mutant clones lead to dysregulated myeloid differentiation (A) UMAP showing the myeloid differentiation trajectory with cells colored by pseudotime score. (B) Top: density plot showing the distribution of *TET2*^WT^ and *TET2*^MUT^ cells through pseudotime in the myeloid lineage. Cells sorted from the total Lin^−^CD34^+^ fluorescence-activated cell sorting (FACS) gate were downsampled to an equal number of cells per sample (*n* = 178 cells from each of the 4 samples). Bottom: density of cells in each cluster along pseudotime. (C) GSEA against hematopoietic signatures comparing *TET2*^MUT^ versus *TET2*^WT^ cells (*n* = 4 *TET2*^MUT^ CH samples) within myeloid lineage clusters. Differential expression analysis was performed accounting for sample and batch effects. Signatures with FDR > 0.05 are colored gray. Positive normalized enrichment scores (NESs) indicate enrichment in mutant cells. LT-HSC, long-term HSC; ST-HSC, short-term HSC. (D) Local regression of AUCell expression scores for HSC and myeloid gene signatures along myeloid pseudotime, comparing *TET2*^WT^ and *TET2*^MUT^ cells. Shading indicates the 95% CI. (E) Volcano plot showing differentially expressed regulons between *TET2*^MUT^ and *TET2*^WT^ cells within the LMPP cycling and early GMP clusters in *TET2*^MUT^ CH samples. FDR-corrected *p* values calculated by linear mixed model test accounting for sample effects. x axis shows the mean of the change in regulon activity (area under the receiver operator curve) calculated within each sample. (F) UMAPs showing activity of the indicated regulons across the hematopoietic landscape within non-CH samples. (G) Local regression of regulon activity through myeloid pseudotime, comparing *TET2*^MUT^ and *TET2*^WT^ cells. Shading indicates the 95% CI. (H) Fitted gene expression values along myeloid pseudotime for the transcription factors shown in (F) and (G) in *TET2*^MUT^ and *TET2*^WT^ cells. Shading indicates the 95% CI. (I) Enrichment of TF motifs within differentially methylated enhancer regions (DMRs) that are hypermethylated in monocytes from *TET2*-mutant CCUS patients,^81^ plotted against the mean change in regulon activity between *TET2*^MUT^ and *TET2*^WT^ cells within the LMPP cycling and early GMP clusters from (E). See also Figure S5.

As *TET2*^MUT^ clonal expansion was most pronounced in the myeloid lineage, we further explored the transcriptional basis of this phenotype in individuals with *TET2*^MUT^ CH. We first compared the density distributions of *TET2*^MUT^ and *TET2*^WT^ cells along the differentiation trajectory from HSCs to mature myeloid cells (Figure 4A). *TET2*^MUT^ cells accumulated, particularly at the progenitor stage, within the cycling LMPP and GMP clusters (Figure 4B).

*TET2*^MUT^ myeloid progenitor expansion might arise due to increased myeloid differentiation from HSCs and/or delayed terminal maturation of mutant myeloid progenitors. To investigate this, we performed gene set enrichment analysis (GSEA) using published HSPC signatures^61^^,^^62^^,^^66^ validated in our dataset (Figure S5A) and examined transcriptional differences between *TET2*^MUT^ and *TET2*^WT^ cells within LMPPs and GMPs. *TET2*^MUT^ progenitors were negatively enriched for HSC signatures (Figure 4C), suggesting decreased retention of stemness programs. Conversely, *TET2*^MUT^ progenitors, particularly GMPs, were enriched for neutrophil progenitor and mature neutrophil/monocyte signatures (Figure 4C). Importantly, these patterns were consistent across multiple individual samples (Figure S5B). Concordantly, CD38 and CD45RA surface protein expression was higher in *TET2*^MUT^ than *TET2*^WT^ LMPPs (Figure S5C), consistent with our previous observations that higher expression of these markers enriches for myeloid potential in LMPPs.^67^ Megakaryocytic-erythroid signatures were negatively enriched, particularly in *TET2*^MUT^ cycling LMPPs (Figure S5D), concordant with their myeloid bias. *TET2*^MUT^ progenitors also upregulated signatures associated with cell cycle (in cycling LMPP), oxidative phosphorylation, cytokine signaling, and innate immune effector function (in GMP) (Figure S5E). Conversely, cell cycle and oxidative phosphorylation signatures were negatively enriched in *TET2*^MUT^ Pro-B/Pre-B cells (Figure S5E), providing a potential explanation for the relative depletion of *TET2*^MUT^ cells in mature B cells (Figure 3G).

To explore these transcriptional alterations throughout the continuum of myeloid differentiation, we compared AUCell scores^82^ for the same HSPC signatures between *TET2*^MUT^ and *TET2*^WT^ cells across myeloid pseudotime. HSC-related genes were downregulated earlier in *TET2*^MUT^ cells, while neutrophil progenitor and monocyte signatures showed premature upregulation in *TET2*^MUT^ cells (Figure 4D). Concordantly, exemplar genes expressed in mature myeloid cells, including *MPO*, *NKG7*, *KLF4*, and *RBM47*, showed premature expression in *TET2*^MUT^ progenitors (Figure S5F). This suggests that early *TET2*^MUT^ lympho-myeloid progenitors retain less HSC and non-myeloid programs, committing more rapidly to myelopoiesis.

To identify potential drivers of dysregulated myeloid differentiation in *TET2*^MUT^ progenitors, we used pySCENIC to identify transcription factors (TFs) and their putative downstream target genes (i.e., regulons). We compared regulon expression between *TET2*^MUT^ and *TET2*^WT^ cells within LMPP and early GMP clusters, where lymphoid and myeloid lineages diverge. The canonical myeloid TFs CEBPD, CEBPA, IRF8, SPI1, and SPIB were more active in *TET2*^MUT^ progenitors (Figure 4E). Conversely, TFs associated with HSC self-renewal (MEIS1, HOXA9, HOXB2, NFYB, and PBX1) and with megakaryocytic-erythroid differentiation (GATA1, TAL1, and KLF1) were less active in *TET2*^MUT^ cells. Regulon activity (Figures 4F and 4G) and TF expression (Figure 4H) of *CEBPA*, *SPI1*, *IRF8*, and *CEBPD* peaked earlier in *TET2*^MUT^ cells along myeloid pseudotime. This quartet of myeloid TFs is required both in early (CEBPA, SPI1, and IRF8) and later (SPI1, IRF8, and CEBPD) stages of myelopoiesis. By contrast, *MEIS1* and its targets were downregulated earlier in *TET2*^MUT^ cells. Interestingly, binding motifs of CEBPA, CEBPD, SPI1, SPIB, and ELF1/2 were also enriched within differentially methylated enhancers in PB granulocytes from patients with *TET2*^MUT^ clonal cytopenia of undetermined significance (CCUS) (Figure 4I).^81^ This suggests a link between altered enhancer methylation and dysregulated myeloid TF activity in *TET2*^MUT^ myeloid cells. Overall, these findings show *TET2*^MUT^ myeloid progenitors are biased toward maturation, with accelerated upregulation of mature transcriptional programs.

### Non-cell-autonomous activation of inflammatory transcriptional programs in CH is attenuated in mutant HSCs

Humans have an estimated 50,000–200,000 HSCs.^83^ In an individual where 1% of HSCs harbor a CH mutation, this represents a 500- to 2,000-fold expansion from a single initiating mutant HSC. Compared with this, changes in clone size downstream of HSCs are modest, implying that the greatest clonal expansion for both *DNMT3A*^MUT^ and *TET2*^MUT^ (CH^MUT^) clones occurred within long-lived HSCs. At least two hypotheses could explain the relative clonal advantage of CH^MUT^ HSCs over WT HSCs (CH^WT^ HSCs): either CH^MUT^ HSCs have a cell-autonomous (intrinsic) competitive advantage and/or CH^WT^ HSCs are at a competitive disadvantage due to cell-extrinsic differences in the CH BM microenvironment.

**Figure 5:**
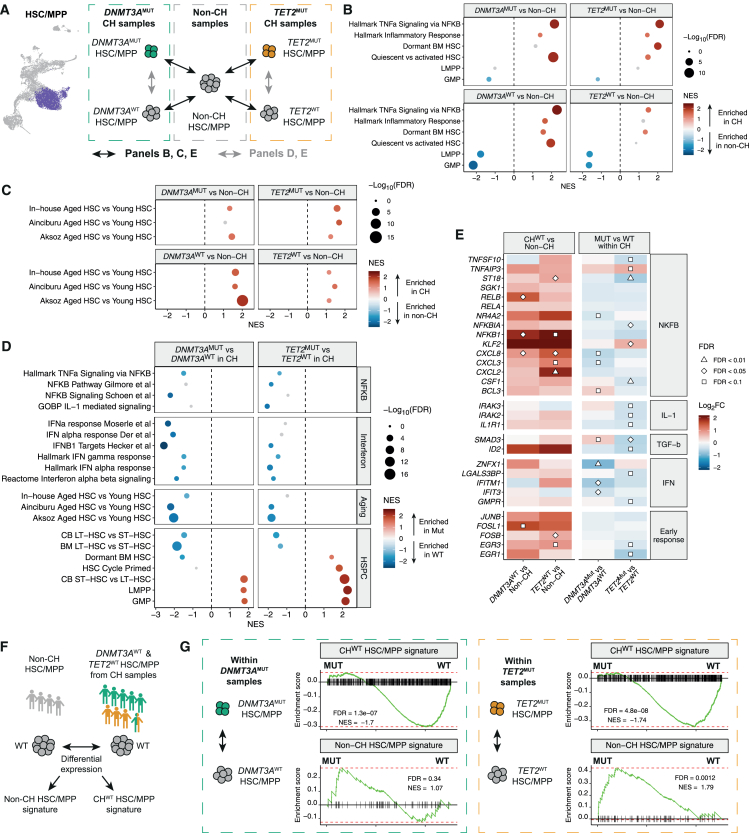
Non-cell-autonomous activation of inflammatory transcriptional programs in clonal hematopoiesis is attenuated in mutant HSCs (A) Strategy for differential gene expression analysis between HSC/MPPs from CH samples and HSC/MPPs from age-matched non-CH samples (black arrows; B, C, and E), and between CH^MUT^ and CH^WT^ HSC/MPPs within CH samples (gray arrows; D and E). (B) GSEA against inflammatory and hematopoietic lineage signatures comparing CH^WT^ or CH^MUT^ HSC/MPPs versus non-CH HSC/MPPs. Differential expression analysis was performed accounting for sample, age, and batch effects. Left: 5 *DNMT3A*^MUT^ samples (*n* = 1,139 *DNMT3A*^WT^ cells, *n* = 409 *DNMT3A*^MUT^ cells) versus 4 non-CH samples (*n* = 1,279 cells). Right: 3 *TET2*^MUT^ samples (*n* = 1,239 *TET2*^WT^ cells, *n* = 222 *TET2*^MUT^ cells) versus 4 non-CH samples (*n* = 1,279 cells). The double-mutant NOC115 sample was excluded. Signatures with FDR > 0.2 are colored gray. Positive NES values indicate enrichment in CH samples. (C) As in (B) but showing GSEA against aged HSC signatures derived from the in-house dataset and from two additional studies comparing aged and young human HSCs.^84^^,^^85^ (D) GSEA against NF-κB, interferon, and hematopoietic signatures comparing *DNMT3A*^MUT^ versus *DNMT3A*^WT^ HSC/MPPs (left) and *TET2*^MUT^ versus *TET2*^WT^ HSC/MPPs (right) within CH samples. Signatures with FDR > 0.2 are colored gray. Positive NES values indicate enrichment in mutant cells. CB, cord blood. (E) Heatmap showing log_2_ fold change in expression of genes related to inflammatory pathways within HSC/MPP. Left: comparison of CH^WT^ versus non-CH cells. Right: comparison of CH^MUT^ versus CH^WT^ cells within CH samples. Symbols represent FDR-corrected *p* values from differential expression testing. (F) Strategy for deriving CH^WT^ and non-CH HSC/MPP signatures. Differential expression analysis was performed between HSC/MPPs from the 4 non-CH samples (*n* = 1,279 cells) and CH^WT^ cells from the 9 CH samples (*n* = 2,622 cells), accounting for sample, age, and batch effects. (G) GSEA enrichment plots for the CH^WT^ HSC/MPP signature (top) and non-CH HSC/MPP signature (bottom), comparing CH^WT^ and CH^MUT^ cells within CH samples. Positive enrichment scores indicate enrichment in mutant cells. See also Figure S6.

To dissect these hypotheses, we first compared gene expression between HSC/MPPs from CH samples (both CH^MUT^ and CH^WT^) and age-matched samples without CH (Figure 5A). Compared with non-CH HSC/MPPs, CH^MUT^ HSC/MPPs showed enrichment of tumor necrosis factor alpha (TNF-α) signaling via nuclear factor κB (NF-κB), inflammatory response, and HSC quiescence signatures (Figure 5B, top). Strikingly, a similar pattern was observed in CH^WT^ versus non-CH HSC/MPPs (Figure 5B, bottom). By contrast, gene sets for LMPP/GMP states were negatively enriched in CH^WT^ HSC/MPPs, suggesting decreased priming toward differentiation. These data suggest both mutant and WT HSC/MPPs in CH individuals are impacted by an inflammatory milieu.

Prior data indicate that aging is associated with chronic inflammation, increased NF-κB signaling, quiescence, and functional decline in HSCs.^86^^,^^87^^,^^88^ To derive human aging-associated transcriptional programs, we performed single-nucleus RNA sequencing (snRNA-seq) on human BM HSPCs and performed differential expression between HSCs from young and aged individuals to define a signature of aged HSCs (Figures S6A and S6B). For further validation, we defined additional aged HSC signatures through re-analysis of two human HSPC single-cell RNA-seq (scRNA-seq) datasets (Figures S6C and S6D; Table S6).^84^^,^^85^ Notably, these aged HSC signatures were enriched in both CH^MUT^ and CH^WT^ HSC/MPPs compared with age-matched non-CH HSC/MPPs (Figure 5C).

We then asked how *DNMT3A* and *TET2* mutations alter gene expression to provide CH^MUT^ HSC/MPPs a fitness advantage within the CH BM environment. Interestingly, TNF-α signaling, NF-κB pathway, interleukin-1 (IL-1) signaling, and interferon (IFN)-α response signatures were all negatively enriched in CH^MUT^ HSC/MPPs compared with their CH^WT^ counterparts (Figure 5D). Furthermore, aged HSC and LT-HSC signatures were also negatively enriched in CH^MUT^ compared with CH^WT^ HSC/MPPs, particularly in individuals with *DNMT3A*^MUT^ CH. These patterns were shared across multiple individual samples, despite some heterogeneity across individuals (Figure S6E). Specific genes associated with interferon, NF-κB, IL-1, transforming growth factor β (TGF-β) signaling, and early response were more highly expressed in CH^WT^ compared with non-CH HSC/MPPs (Figure 5E, left). Conversely, many of the same genes were expressed at lower levels in CH^MUT^ compared with CH^WT^ HSC/MPPs (Figure 5E, right). Interestingly, *TNFAIP3*, encoding A20, which inhibits NF-κB activation, was upregulated in *TET2*^MUT^ HSC/MPP. By contrast, signatures of short-term HSC (ST-HSC), LMPP, and GMP were enriched in CH^MUT^ compared with CH^WT^ HSC/MPPs (Figure 5D), suggesting an inverse relationship between molecular programs underlying inflammation and aging in contrast to lympho-myeloid differentiation priming. Consistent with reduced quiescence, CH^MUT^ HSC/MPPs were enriched for pathways related to mitosis, cell migration, and signaling, particularly in *TET2*^MUT^ cells (Figure S6F). Furthermore, *TET2*^MUT^ HSC/MPPs had greater RNA content, were larger, more granular, and had lower CD49f protein expression than *TET2*^WT^ cells (Figure S6G).

Collectively, these data suggest CH^MUT^ HSC/MPPs have an altered transcriptional response to the inflammatory CH environment. To generate molecular signatures that capture differences between the CH and non-CH environment, we compared gene expression profiles of CH^WT^ to non-CH HSC/MPPs (Figures 5F and S6H). Genes upregulated in CH^WT^ HSC/MPPs were enriched for TNF-α via NF-κB, HSC quiescence, and aging signatures (Figure S6I). We then asked whether *DNMT3A*^MUT^ and *TET2*^MUT^ HSC/MPPs were impacted by the CH environment differently than CH^WT^ HSC/MPPs. Indeed, both *DNMT3A*^MUT^ and *TET2*^MUT^ HSC/MPPs were negatively enriched for the CH^WT^ HSC/MPP signature (Figure 5G top), while *TET2*^MUT^ HSC/MPPs were positively enriched for the non-CH HSC/MPP signature (Figure 5G bottom), consistent with our findings above.

In summary, our data indicate that, compared with their CH^WT^ counterparts, CH^MUT^ HSC/MPPs are transcriptionally more similar to non-CH HSC/MPPs and have an attenuated transcriptional response to aging and inflammation.

### The effects of *DNMT3A* and *TET2* mutations are most prominent in a transcriptionally distinct subset of HSCs

**Figure 6:**
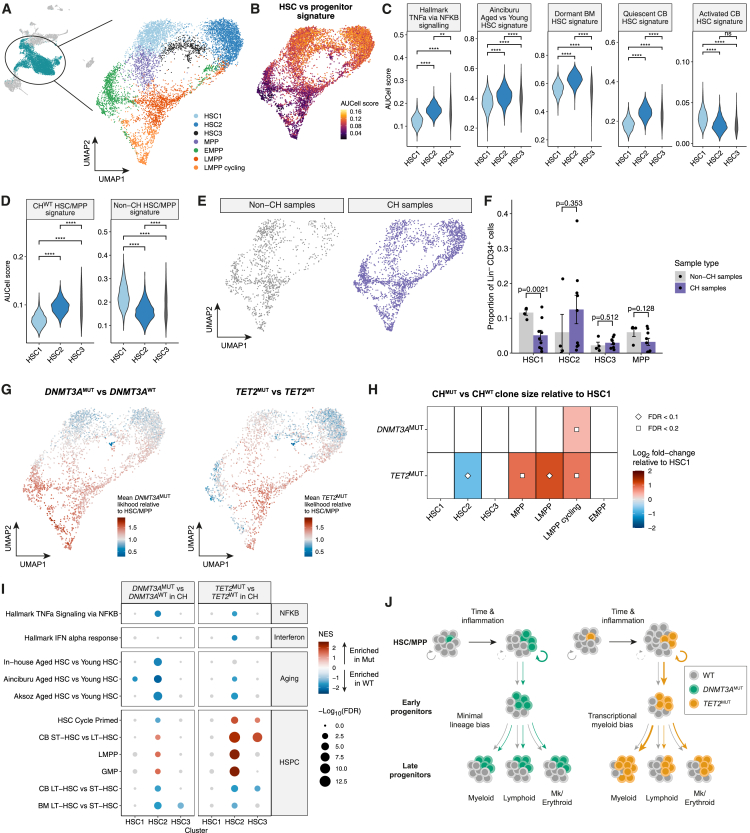
The effects of *DNMT3A* and *TET2* mutations are most prominent in a transcriptionally distinct subset of HSCs (A) UMAP of 8,059 cells from the HSC/MPP, EMPP, LMPP, and LMPP cycling clusters after feature weight derivation with the SAM algorithm, colored by cluster annotation. (B) UMAP superimposed with AUCell scores for a signature of genes differentially expressed between HSCs and progenitors.^66^ (C) AUCell scores for TNF-α via NF-κB, HSC aging, dormant BM HSC,^61^ and quiescent versus activated CB HSC signatures,^89^ comparing the HSC clusters. *p* values calculated by unpaired t test. The area of each violin is proportional to cell number. (D) AUCell scores for the CH^WT^ and non-CH HSC/MPP signatures, comparing the HSC clusters. *p* values calculated by unpaired t test. (E) UMAP embeddings showing cells from non-CH and CH samples. (F) Quantification of the size of each HSC/MPP cluster as a proportion of Lin^−^CD34^+^ cells, comparing CH and non-CH samples. Only cells sorted from the total Lin^−^CD34^+^ FACS gate were included. Data are represented as mean ± SEM. Each dot represents a sample. *p* values calculated by unpaired t test. (G) UMAPs of cells from *DNMT3A*^MUT^ CH samples (left) and *TET2*^MUT^ CH samples (right) colored by the mean likelihood of cells being in the mutant clone relative the average in the HSC/MPP. The mean relative likelihood across all samples analyzed is shown (*n* = 6 *DNMT3A*^MUT^ samples; *n* = 4 *TET2*^MUT^ samples). A relative likelihood ≥1 indicates that the probability of a cell being mutant is greater than the average for the HSC/MPP. (H) Heatmap of Log_2_FC in abundance of mutant clones relative to the HSC1 cluster (from scCODA). Only significant results at FDR < 0.2 are shown; nonsignificant differences plotted as white. (I) GSEA against NF-κB, interferon, aging, and hematopoietic signatures comparing *DNMT3A*^MUT^ versus *DNMT3A*^WT^ HSC/MPPs (left) and *TET2*^MUT^ versus *TET2*^WT^ HSC/MPPs (right) within CH samples. Signatures with FDR > 0.05 are colored gray. Positive NES values indicate enrichment in mutant cells. (J) Model of *DNMT3A*^MUT^ and *TET2*^MUT^ clonal expansion. Inflammation impairs the function of CH^WT^ HSCs, but mutant HSCs are less affected, leading to clonal expansion over time (circular arrows). Downstream of the HSC, both *DNMT3A*^MUT^ and *TET2*^MUT^ clones expand moderately in early progenitors (linear arrows). In later differentiation, *DNMT3A*^MUT^ clone size is largely maintained, but *TET2*^MUT^ clones expand further and have a myeloid bias. ^∗^*p* < 0.05, ^∗∗^*p* < 0.01, ^∗∗∗^*p* < 0.001, ^∗∗∗∗^*p* < 0.0001. See also Figure S6.

Transcriptional differences between CH and non-CH HSC/MPPs warranted a more granular exploration of HSC/MPP heterogeneity. Thus, we subclustered HSC/MPPs and the earliest progenitors from CH and non-CH samples (Figures 6A and S6J) using self-assembling manifolds (SAMs), an unsupervised approach to prioritize biologically relevant features among comparatively homogeneous cells, which has previously been applied to human HSC/MPPs.^89^^,^^90^^,^^91^ This identified three distinct HSC clusters (HSC1–3), of which HSC1 and HSC2 contained most cells. All three HSC clusters showed transcriptional and immunophenotypic features of HSCs, although HSC3 was immunophenotypically more like MPPs (Figures 6B, S6K, and S6L).

Next, we asked whether the HSC clusters were transcriptionally distinct with respect to gene signatures of inflammation and aging (Figure 6C). Interestingly, HSC2 expressed higher levels of TNF-α/NF-κB signaling, aged HSC, and quiescence signatures,^61^^,^^89^ and lower levels of the activated HSC signature. Concordant with their more quiescent transcriptional profile, HSC2 expressed fewer genes than HSC1 (Figure S6M). HSC3 exhibited a more heterogeneous expression profile, intermediate to HSC1 and HSC2. This was mirrored by the expression of exemplar genes associated with inflammatory signaling, quiescence, and cell cycle (Figure S6N). For example, HSC2 expressed lower levels of *CDK6*, which promotes exit from quiescence in LT-HSCs,^92^ and higher levels of *GPRC5C*, which marks dormant human BM HSCs.^61^ Interestingly, though HSC1 cells showed higher expression of genes that promote exit from quiescence, they also showed higher expression of genes and TF regulons implicated in HSC self-renewal (Figures S6N and S6O). Taken together, this suggests the HSC2 cluster has a transcriptional phenotype reflecting greater NF-κB pathway activity, increased quiescence, and decreased expression of TFs that support self-renewal.

Interestingly, HSC2 cells expressed higher levels of the CH^WT^ HSC/MPP signature and lower levels of the non-CH HSC/MPP signature (Figure 6D). To determine whether the heightened expression of inflammatory signatures in CH HSC/MPPs correlated with differences in HSC cluster composition, we compared the frequency of cells in the different HSC and MPP clusters. Interestingly, the HSC1 cluster was smaller in CH samples and the HSC2 cluster was larger, though the latter did not reach statistical significance (Figures 6E and 6F). This suggests differences in HSC cluster composition between CH and non-CH samples may provide a basis for the heightened transcriptional response to inflammation in CH.

The co-existence of distinct HSC states prompted us to ask whether CH mutations exerted context-specific transcriptional effects within the heterogeneous HSC compartment. Specifically, we sought to identify the cellular basis of attenuated expression of inflammatory and aging signatures in CH^MUT^ HSCs. One possible hypothesis is that CH^MUT^ cells are enriched in HSC1 compared with the HSC2 cluster. Examining the ratio of CH^MUT^ to CH^WT^ cells showed *TET2*^MUT^ cells were significantly depleted in HSC2 relative to HSC1, with clone size being smaller in 3 out of 4 *TET2*^MUT^ samples (Figures 6G, 6H, S6P, and S6Q). However, there was no significant difference for *DNMT3A*^MUT^ clones. Thus, depletion of mutant cells in HSC2 may partly account for the attenuated effect of inflammatory and aging programs in *TET2*^MUT^ HSCs.

A second, not mutually exclusive hypothesis, is that the transcriptional profiles of different HSC clusters could be differently modified by CH mutations. Specifically within HSC2, *DNMT3A*^MUT^ and *TET2*^MUT^ cells were negatively enriched for TNF-α signaling via NF-κB, aged HSC, and LT-HSC signatures, but positively enriched for ST-HSC, LMPP, and GMP signatures, relative to CH^WT^ HSCs (Figure 6I). Some of these differences were shared in the HSC1 and HSC3 clusters but were less marked. These data support the hypothesis that *DNMT3A* and *TET2* mutations, either directly or indirectly, attenuate expression of transcriptional programs related to inflammatory signaling and aging while promoting lympho-myeloid differentiation programs, principally in the HSC2 cluster.

## Discussion

The first detailed single-cell examination of CH from older individuals with unperturbed hematopoiesis has uncovered new insights into the cell-intrinsic and cell-extrinsic consequences of CH mutations in humans. Our observations reflect the outcome of decades-long clonal competition within the human BM. The high-fidelity genotyping achieved with TARGET-seq+ ensures more than 90% of cells are genotyped, compared with ∼20% with droplet-based approaches,^54^ and is coupled to high-quality scRNA-seq data. Therefore, we can more confidently assign transcriptomes to either CH^WT^ or CH^MUT^ cells and discriminate clone-specific transcriptional programs.

Our data show that clones harboring heterozygous *DNMT3A* or *TET2* mutations follow a normal differentiation trajectory, although some lineage bias is observed. Quantitatively, most of the steady-state fitness advantage of *DNMT3A*^MUT^ and *TET2*^MUT^ clones occurs at the HSC/MPP level (Figure 6J). *DNMT3A*^MUT^ clones show no consistent lineage bias, in contrast to data from myeloma remission samples, in which *DNMT3A*^R882^-mutant CH cells were biased toward the megakaryocytic-erythroid lineage.^54^ This discrepancy may either be due to true biological differences between *DNMT3A*^R882^ and other *DNMT3A* mutations or to technical differences between TARGET-seq+ and droplet-based methods, the latter of which rely on RNA expression for genotyping. *TET2*^MUT^ clones further expand 2- to 4-fold within the LMPP/GMP. Immature *TET2*^MUT^ lympho-myeloid progenitors retain less HSC and non-myeloid transcriptional programs while showing premature upregulation of mature programs, possibly due to premature activity of myeloid transcription factors. These data are concordant with recent findings that *TET2*^MUT^ HSPCs show exacerbated production of aberrant neutrophils in a cord blood knockout model.^56^

Importantly, our data support the notion of a more inflammatory environment in CH. Chronic inflammatory stimuli impair HSC self-renewal and mimic changes observed in aged mice.^93^^,^^94^^,^^95^^,^^96^ Prior murine studies show that *Tet2*^*−/−*^, *Dnmt3a*^*−/−*^, and *Dnmt3a*^R878H/+^ HSPCs exhibit clonal advantage in inflammatory environments.^45^^,^^46^^,^^47^^,^^48^^,^^49^^,^^50^^,^^97^ Intestinal bacterial translocation,^48^ chronic mycobacterial infection^45^ or exposure to various cytokines, including IL-1,^49^ IL-6,^47^^,^^48^ TNF-α,^46^^,^^97^ and IFN-γ,^45^ promote clonal advantage of mutant HSPCs. Furthermore, Tet2 directly represses the pro-inflammatory cytokine IL-6.^32^ However, no study has yet fully elucidated the differential impact of inflammation on co-existing WT and CH^MUT^ HSCs in native hematopoiesis. Furthermore, animal models may not adequately replicate human CH, where clones are exposed to changing environments over decades under heterogeneous selective pressures.

Our results suggest that CH mutations, directly or indirectly, attenuate the deleterious response to inflammation within HSCs. In agreement with our findings, data from zebrafish show that subclonal CH mutations lead to the expression of pro-inflammatory genes in mutant mature myeloid cells but anti-inflammatory genes in mutant HSPCs, providing them with a relative fitness advantage.^98^ Furthermore, in mice, expansion of *Tet2*^*+/−*^ subclones exposed to IL-1 is associated with attenuated downregulation of HSC self-renewal genes compared with WT HSCs,^49^ whereas clonal advantage of *Dnmt3a*^R878H^ HSPCs correlates with reduced activation of necroptosis in response to TNF-α challenge.^97^ Together, these data suggest a model whereby the CH^MUT^ HSC population gains an advantage over the vastly more numerous non-mutant HSCs through differential impact of inflammation on their function (Figure 6J).

Further work is now needed to define the diverse drivers of inflammation in humans and the mechanisms by which human CH^MUT^ HSCs resist the detrimental effects of inflammation. The inflammatory drivers are likely to be heterogeneous and vary over time. Nevertheless, there may be common pathways of gene regulation downstream of multiple inflammatory signals. Examination of the BM niche in individuals with CH would thus be helpful. This, if combined with functional analyses and inferred rates of clonal expansion from the same individuals, may help to identify the most potent inflammatory drivers of CH^MUT^ clonal selection. These datasets could ultimately lead to therapies that diminish the inflammatory selective pressures or the ability of CH^MUT^ HSCs to resist the deleterious effects of inflammation and aging.

### Limitations of the study

While we provide a hypothesis for clonal selection in human CH, functional experiments are required to define those pathways causative of clonal expansion, although prior functional murine studies support our findings.^32^^,^^45^^,^^46^^,^^47^^,^^49^^,^^97^ Second, additional functions of DNMT3A and TET2 beyond transcriptional regulation^30^^,^^31^^,^^32^^,^^33^^,^^34^^,^^35^^,^^36^^,^^37^ may also play important roles in clonal advantage. Third, our findings do not fully reveal the mechanistic link between altered DNMT3A/TET2 function and downstream pathways. Future studies integrating single-cell genotyping with chromatin accessibility^99^ and DNA binding in primary CH samples may elucidate the epigenetic basis for the observed transcriptional changes.

Since osteoarthritis is associated with low-grade chronic inflammation,^100^ the samples analyzed may have been more impacted by inflammation than in individuals without osteoarthritis. However, since both CH and non-CH individuals had osteoarthritis, all our analyses are controlled for this effect.

Importantly, we appreciate that our conclusions depend on the accuracy of single-cell genotyping. All single-cell genotyping approaches are affected by ADO, which varies considerably depending on the method. ADO can result in significant mis-genotyping, impacting downstream analyses, especially when clones are small. Importantly, TARGET-seq+ yields lower ADO rates compared with droplet-based technologies. In our study, cells in which ADO of the mutant allele occurred could only be identified at four loci where a heterozygous germline SNP was present in the amplicon (Figures S3E–S3H). We estimate the ADO rate to be 5%–15%, depending on the locus and quantification method used (STAR Methods). For the other 8 mutations, the inferred ADO rate was approximately 5%–13.5% (Figures S3I and S3J). This would have resulted in 5%–13.5% of mutant cells being called WT. We have tested the impact of this ADO rate on downstream analyses by performing simulations in which we deliberately mis-classified 10% of mutant HSC/MPP cells as WT. The GSEA results obtained were highly concordant with the original analysis, albeit with reduced enrichment scores and significance (Table S6). Overall, this suggests that modest ADO rates lead to a subtle “dilution” of the apparent differences between CH^WT^ and CH^MUT^ cells, without altering our conclusions.

## STAR★Methods

### Key resources table

**Table:** 

REAGENT or RESOURCE	SOURCE	IDENTIFIER
**Antibodies**		

BV421 anti-human CD38 (clone HIT2)	BioLegend	Cat# 303526; RRID:AB_10983072
BV605 anti-human CD10 (clone HI10a)	BioLegend	Cat# 312222; RRID:AB_2562157
BV650 anti-human CD49f (clone GoH3)	BioLegend	Cat# 313629; RRID:AB_2686989
BV785 anti-human CD117 (clone 104D2)	BioLegend	Cat# 313238; RRID:AB_2629837
BB515 anti-human CD45RA (clone HI100)	BD	Cat# 564552; RRID:AB_2738841
PE anti-human CD123 (clone 6H6)	BioLegend	Cat# 306006; RRID:AB_314580
PE/Dazzle 594 anti-human CD49f (clone GoH3)	BioLegend	Cat# 313626; RRID:AB_2616782
PE/Cy7 anti-human CD90 (clone 5E10)	BioLegend	Cat# 328124; RRID:AB_2561693
APC anti-human CD34 (clone 581)	BioLegend	Cat# 343510; RRID:AB_1877153
PE/Dazzle 594 anti-human CD117 (clone 104D2)	BioLegend	Cat# 313226; RRID:AB_2566213
PE/Cy5 anti-human CD2 (clone RPA-2.10)	BioLegend	Cat# 300210; RRID:AB_314034
PE/Cy5 anti-human CD3 (clone HIT3a)	BioLegend	Cat# 300310; RRID:AB_314046
PE/Cy5 anti-human CD4 (clone RPA-T4)	BioLegend	Cat# 300510; RRID:AB_314078
PE/Cy5 anti-human CD8a (clone RPA-T8)	BioLegend	Cat# 301010; RRID:AB_314128
PE/Cy5 anti-human CD11b (clone ICRF44)	BioLegend	Cat# 301308; RRID:AB_314159
PE/Cy5 anti-human CD14 (clone 61D3)	eBioscience	Cat# 15-0149-42; RRID:AB_2573058
PE/Cy5 anti-human CD19 (clone HIB19)	BioLegend	Cat# 302210; RRID:AB_314240
PE/Cy5 anti-human CD20 (clone 2H7)	BioLegend	Cat# 302308; RRID:AB_314256
PE/Cy5 anti-human CD56 (clone MEM188)	BioLegend	Cat# 304608; RRID:AB_314450
PE/Cy5 anti-human CD235ab (clone HIR2)	BioLegend	Cat# 306606; RRID:AB_314623
FITC anti-human CD45RA (clone HI100)	BD	Cat# 555488; RRID:AB_395879
PE anti-human CD90 (clone 5E10)	BD	Cat# 555596; RRID:AB_395970
BV711 anti-human CD19 (clone SJ25C1)	BD	Cat# 563036; RRID:AB_2737968
PE/Cy5 anti-human CD49f (clone GoH3)	BD	Cat# 551129; RRID:AB_394062
APC anti-human CD271 (clone ME20.4-1.H4)	Miltenyi	Cat# 130-113-418; RRID:AB_2733363
APC/Cy7 anti-human CD34 (clone 581)	BD	Custom-made
PE/Cy7 anti-human CD38 (clone HB7)	BD	Cat# 335790; RRID:AB_399969
Alexa Fluor 700 anti-human CD10 (clone HI10a)	BD	Cat# 563509; RRID:AB_2738247
BV605 anti-human CD14 (clone M5E2)	BD	Cat# 564054; RRID:AB_2687593
V500 anti-human CD45 (clone HI30)	BD	Cat# 560777; RRID:AB_1937324
BV421 anti-human CD33 (clone WM53)	BioLegend	Cat# 303416; RRID:AB_2561690

**Chemicals, peptides, and recombinant proteins**		

RPMI-1640	Gibco	Cat# 21875034
IMDM	Gibco	Cat# 21056023
DPBS	ThermoFisher	Cat# 14190169
Fetal Bovine Serum	Sigma-Aldrich	Cat# F7524
Heparin sodium	Sigma-Aldrich	Cat# H3149
D-(+)-Glucose (dextrose)	Sigma-Aldrich	Cat# G7528
Citric Acid, trisodium salt, dihydrate	Sigma-Aldrich	Cat# C3434
Citric Acid, anhydrous	Sigma-Aldrich	Cat# C0759
Penicillin-Streptomycin (10,000 U/mL)	Gibco	Cat# 15140122
DNase I	Roche	Cat# 11284932001
Ficoll-Paque PLUS	GE Healthcare	Cat# 17-1440-03
Propidium iodide	Biolegend	Cat# 421301
Propidium iodide	BD	Cat# 5564463
7-AAD	Biolegend	Cat# 420404
SDS 10%	Sigma-Aldrich	Cat# L4509
Triton X-100	Sigma-Aldrich	Cat# T8787
dNTPs (10 mM each)	Thermo Scientific	Cat# R0193
Poly-ethylene Glycol 8000 (40% solution)	Sigma-Aldrich	Cat# P1458
Recombinant RNAse Inhibitor (40 U/μL)	Takara	Cat# 2313B
Protease	Qiagen	Cat# 19155
Nuclease-free water	Invitrogen	Cat# AM9937
Tris-HCl 1M pH 8.0	Thermo Scientific	Cat# 15893661
NaCl 5M	Invitrogen	Cat# AM9760G
MgCl2 1M	Invitrogen	Cat# AM9530G
GTP Solution, Tris buffered	Thermo Scientific	Cat# R1461
Dithiothreitol (DTT), 0.1M Solution	Thermo Scientific	Cat# 707265ML
UltraPure Agarose	Invitrogen	Cat# 16500-500
Ethidium Bromide Solution	Invitrogen	Cat# 15585-011
MseI restriction enzyme	New England Biolabs	Cat# R0525S
ddPCR Supermix for Probes (No dUTP)	Bio-Rad	Cat# 186-3023

**Critical commercial assays**		

CD34 MicroBead kit	Miltenyi	Cat# 130-046-703
LS Columns	Miltenyi	Cat# 130-042-401
MycoAlert Mycoplasma Detection Kit	Lonza	Cat# LT07-218
CompBeads	BD Biosciences	Cat# 552843
DNeasy Blood & Tissue Kit	Qiagen	Cat# 69506
KAPA HyperPlus Kit	Roche	Cat# 07962428001
KAPA Dual-Indexed Adapter Set	Roche	Cat# 08278555702
HyperCap Target Enrichment Kit	Roche	Cat# 08286345001
HyperCap Bead Kit	Roche	Cat# 08286400001
SeqCap EZ Prime Choice Library (custom probes)	Roche	Cat# 08247480001
AMPure XP Beads	Beckman Coulter	Cat# A63881
Qubit dsDNA HS Assay Kit	Invitrogen	Cat# Q32854
ERCC RNA Spike-In Mix	Invitrogen	4456740
Maxima H Minus Reverse Transcriptase (200 U/μL)	Thermo Scientific	Cat# EP0753
KAPA HiFi HotStart ReadyMix	Roche	Cat# 07958935001
KAPA 2G Robust HS Ready Mix	Sigma-Aldrich	Cat# KK5702
FastStart High Fidelity PCR System, dNTPack	Sigma-Aldrich	Cat# 4738292001
Nextera XT DNA Library Preparation Kit	Illumina	Cat# FC-131-1096
Nextera XT Index Kit Set v2 Set A	Illumina	Cat# FC-131-2001
Nextera XT Index Kit Set v2 Set C	Illumina	Cat# FC-131-2003
Access Array Barcode Library for Illumina Sequencers-384, Single Direction	Fluidigm	Cat# 100-4876
High Sensitivity NGS Fragment Analysis Kit (1 bp–6,000 bp)	Agilent	Cat# DNF-474-0500
Agilent High Sensitivity DNA Kit	Agilent	Cat# 5067-4626
Agilent Tapestation HS D1000 ScreenTape	Agilent	Cat# 5067-5583
Agilent Tapestation HS D1000 Reagents	Agilent	Cat# 5067-5584

**Deposited data**		

Targeted DNA sequencing, raw data	This paper	EGA: EGAS00001007358
TARGET-seq+ single-cell RNA sequencing, raw data	This paper	EGA: EGAS00001007358
TARGET-seq+ single-cell genotyping, raw data	This paper	EGA: EGAS00001007358
TARGET-seq+ single-cell RNA sequencing, processed raw counts	This paper	Figshare: https://doi.org/10.25446/oxford.23576379
TARGET-seq+ single-cell genotyping data, processed allelic counts	This paper	Figshare: https://doi.org/10.25446/oxford.23576421
TARGET-seq+ single-cell metadata and genotypes	This paper	Figshare: https://doi.org/10.25446/oxford.23576262
In-house aged and young bone marrow snRNA-seq, raw data	This paper	GEO: GSE235646
Ainciburu aged and young bone marrow scRNA-seq, raw data	Ainciburu et al.^84^	GEO: GSE180298
GENCODE human gene annotation (v38)	GENCODE project	https://www.gencodegenes.org/human/release_38.html
cisTarget databases (Homo sapiens - hg38 - mc_v10_clust)	Aerts lab; Van de Sande et al.^101^	https://resources.aertslab.org/cistarget/databases/

**Experimental models: Cell lines**		

JURKAT	ATCC	RRID:CVCL_0367

**Oligonucleotides**		

OligodT-ISPCR (HPLC purification): AAGCAGTGGTATCAACGCAGAGTA CTTTTTTTTTTTTTTTTTTTTTTTTTTT TTTVN	Picelli et al.^102^	N/A
Barcoded oligodT-ISPCR primers	Biomers (design: Rodriguez-Meira et al.^59^)	N/A
TSO-LNA (RNase Free HPLC purification): AAGCAGTGGTAT CAACGCAGAGTACATrGrG+G	IDT (design: Picelli et al.^102^)	N/A
ISPCR primer (HPLC purification): AAGCAGTGGTATCAACGCAGAGT	IDT (design: Picelli et al.^102^)	N/A
See Table S4 for target-specific genotyping primers used in the pre-amplification step (RNase Free HPLC purification)	IDT (design: this paper)	N/A
See Table S4 for target-specific nested barcoded genotyping primers used in the PCR1 barcoding step (Standard desalting)	IDT (design: this paper)	N/A
See Table S4 for custom transcriptome i5 index primers (HPLC purification)	IDT (design: this paper)	N/A
See Table S4 for the list of probes used for ddPCR (HPLC purification)	Biomers (design: this paper)	N/A
See Table S4 for the list of primers used for ddPCR (Standard desalting)	IDT (design: this paper)	N/A
P5-SEQ primer (PAGE purification): GCCTGTCCGCGGAAGCAGTGGTA TCAACGCAGAGTTGC^∗^T	Rodriguez-Meira et al.^59^	N/A
I5-SEQ primer (PAGE purification): AGCAACTCTGCGTTGATACCACT GCTTCCGCGGACAGG^∗^C	IDT (design: this paper)	N/A
LCS1 sequencing primer (HPLC purified): GGCGACCACCGAGATCTACACTGACG ACATGGTTCTACA	IDT	N/A
CS2 sequencing primer (HPLC purified): T+AC+GGT+AGCAGAGACTTGGTCT	IDT	N/A
CS2rc sequencing primer (HPLC purified): A+GAC+CA+AGTCTCTGCTACCGTA	IDT	N/A

**Software and algorithms**		

Bcl2fastq (v2.20)	Illumina	https://support.illumina.com/sequencing/sequencing_software/bcl2fastq-conversion-software.html
Python (v3)	Python Software Foundation	https://www.python.org
CGAT-core	Bioconda	https://github.com/cgat-developers/cgat-core
Burrows-Wheeler aligner (v0.7.17)	Li and Durbin^103^	https://bio-bwa.sourceforge.net/
GATK (v4.1.2.0)	Broad Institute	https://software.broadinstitute.org/gatk/
Picard (v2.21.3)	Broad Institute	https://broadinstitute.github.io/picard/
VarDictJava (v1.7.0)	Lai et al.^104^	https://github.com/AstraZeneca-NGS/VarDictJava
Bcftools (v1.9)	HTSlib	https://samtools.github.io/bcftools/
Annovar (v20191024)	Yang et al.^105^	https://annovar.openbioinformatics.org/
Integrative Genomics Viewer (IGV; v2.14.1)	Broad Institute	https://software.broadinstitute.org/software/igv/
Primer3Plus	Untergasser et al.^106^	https://www.primer3plus.com
Primer-BLAST	National Library of Medicine	https://www.ncbi.nlm.nih.gov/tools/primer-blast/
TARGET-seq genotyping pipeline	Github; Rodriguez-Meira et al.^59^	https://github.com/albarmeira/TARGET-seq
infSCITE	Github	https://github.com/cbg-ethz/infSCITE
Samtools	HTSlib	http://www.htslib.org/download/
Cutadapt (v3.4)	Bioconda	https://cutadapt.readthedocs.io/en/stable/
FastQC (v0.11.9)	Bioconda	https://www.bioinformatics.babraham.ac.uk/projects/fastqc/
MultiQC (v1.11)	Bioconda	https://multiqc.info
STAR (v2.7.10a)	Github	https://github.com/alexdobin/STAR
R (v4.2.1)	R-Project	https://www.r-project.org
ggplot2 (v3.3.6)	CRAN	https://ggplot2.tidyverse.org
SingCellaR (v1.2.1)	Github	https://github.com/supatt-lab/SingCellaR
Scran (v1.24.0)	Bioconductor	https://bioconductor.org/packages/release/bioc/html/scran.html
M3Drop (v1.22.0)	Bioconductor	https://www.bioconductor.org/packages/release/bioc/html/M3Drop.html
Harmony (v0.1.0)	CRAN	https://github.com/immunogenomics/harmony
variancePartition (v1.26.0)	Bioconductor	https://bioconductor.org/packages/release/bioc/html/variancePartition.html
fgsea (v1.22.0)	Bioconductor	https://bioconductor.org/packages/release/bioc/html/fgsea.html
AUCell (v1.18.1)	Bioconductor	https://bioconductor.org/packages/release/bioc/html/AUCell.html
Scanpy (v1.9.1)	Conda-forge	https://scanpy.readthedocs.io/en/stable/
MELD (v1.0.0)	PyPI	https://github.com/KrishnaswamyLab/MELD
pySCENIC (v0.12.0)	PyPI	https://github.com/aertslab/pySCENIC
FlowJo (v10.8.1)	BD	https://www.bdbiosciences.com/en-gb/products/software/flowjo-v10-software
BioRender	BioRender	https://biorender.com
Custom code for data analysis	This study	N/A

**Other**		

12.5 mL GRIPTIP, Sterile, Filter	INTEGRA Biosciences	Cat# 6455
High Volume MANTIS Chip	FORMULATRIX	Cat# MCHVSMR6

### Resource availability

#### Lead contact

Further information and requests for resources and reagents should be directed to and will be fulfilled by the lead contact, Paresh Vyas (paresh.vyas@imm.ox.ac.uk).

#### Materials availability

The list of all oligo sequences designed in this study and used for single-cell genotyping can be found in Table S4. These include both target-specific oligos used in the PCR after reverse transcription, and nested barcoded target-specific oligos used in genotyping PCR1. Barcoded oligodT-ISPCR primers were kindly provided by Prof. Adam Mead and Dr. Alba Rodriguez-Meira, and the sequences are listed in Table S4.

#### Data and code availability

• Raw targeted DNA sequencing data, TARGET-seq+ scRNA-seq, and TARGET-seq+ single-cell genotyping data have been deposited at European Genome-Phenome Archive (EGA: EGAS00001007358) in order to comply with ethical approvals and are available as of the date of publication. Processed TARGET-seq+ scRNA-seq, single-cell genotyping, and metadata are available through Figshare. Single-nucleus RNA-seq data for the in-house aged and young bone marrow dataset have been deposited in GEO (GEO: GSE235646). Accession numbers are listed in the key resources table.
• This paper does not report original code.
• Any additional information required to reanalyze the data reported in this paper is available from the lead contact upon request.

### Experimental model and subject details

#### Cell culture

JURKAT human cell line was cultured in RPMI-1640 medium (Cat# 21875034, Gibco) supplemented with 10% FBS and 1% V/V Pen-Strep (Cat# 15140122, Gibco). Cells were regularly screened for Mycoplasma contamination using the MycoAlert Mycoplasma Detection Kit (Cat# LT07-218). Cells were passaged every 2-3 days and seeded at approximately 500,000 cells/mL. Cell lines were kept in a CO_2_ incubator at 37^°^C.

#### Patient samples

Patient samples were collected from individuals undergoing elective total hip replacement (THR) surgery at the Nuffield Orthopaedic Centre, Oxford, under the Mechanisms of Age-Related Clonal Haematopoiesis (MARCH) Study. Written informed consent was obtained from all participants in accordance with the Declaration of Helsinki. This study was approved by the Yorkshire & The Humber - Bradford Leeds Research Ethics Committee (NHS REC Ref: 17/YH/0382). Exclusion criteria were: History of rheumatoid arthritis or other inflammatory arthritis, history of septic arthritis in the limb undergoing surgery, history of hematological cancer, bisphosphonate use, and oral steroid use. Patient characteristics are summarized in Table S1.

For the multi-ome analysis in young versus aged human bone marrow, bone marrow cells from young donors (26-year-old female and 24-year-old male) were purchased from Lonza, while bone marrow samples from aged donors (70 and 77-year-old females) undergoing hip replacement surgery were collected at the Traumatology and Orthopedics Hospital Lomas Verdes (IMSS), Mexico. These elderly donors were confirmed to have no dysplasia of any hematopoietic lineages by histological and CBC analysis.^107^ Ethical approval was obtained from the Institutional Review Board (R-2012-785-092). Patient consent was obtained verbally, and as determined by the Institutional Ethical Board.

### Method details

#### Sample collection and processing

Trabecular bone fragments and bone marrow aspirates were obtained from the femoral canal and collected in 10 mL anticoagulated buffer containing acid-citrate-dextrose, heparin sodium and DNase. Samples of peripheral blood were collected in EDTA vacutainers.

All samples were processed within 24 hours of collection. Peripheral blood and bone marrow aspirate samples were diluted 1:1 in RPMI-1640 (Gibco) and filtered through a 70 μm cell strainer. Trabecular bone samples were manually fragmented with scissors and washed thoroughly in RPMI media with DNase to collect trabecular marrow, which was then filtered through a 70 μm cell strainer to obtain a single cell suspension and combined with the bone marrow aspirate. Mononuclear cells were then isolated by Ficoll density gradient separation (Sigma-Aldrich). For some samples, bone marrow CD34^+^ cells were purified using a CD34 MicroBead kit and MACS separation columns (Miltenyi Biotec), according to the manufacturer’s instructions. Unseparated MNCs, CD34-enriched and CD34-depleted fractions were frozen in 90% fetal bovine serum (FBS, Sigma-Aldrich) with 10% dimethyl sulfoxide (DMSO) and stored in liquid nitrogen until further use.

Peripheral blood granulocytic cell pellets isolated by Ficoll density gradient centrifugation were frozen for DNA sequencing analysis of mutations in peripheral blood.

#### Targeted DNA sequencing

##### Library preparation and sequencing

Targeted DNA sequencing was performed on bone marrow MNCs and peripheral blood granulocytic DNA samples. Pre-capture DNA libraries were prepared using the KAPA HyperPlus protocol (Roche). 100 ng of genomic DNA was fragmented by enzymatic fragmentation. Following end repair and A-tailing, adapter ligation was performed using KAPA dual-indexed adapters (Roche). Library cleanup and double-sided size selection was performed using Agencourt AMPure XP beads (Beckman Coulter) to obtain fragments of ∼320 bp. Libraries were amplified by ligation-mediated PCR for 6 cycles using a KAPA HiFi HotStart high-fidelity DNA polymerase (Roche) and purified using AMPure XP beads.

Targeted capture was performed using a custom pool of biotinylated capture probes (SeqCap EZ Prime Choice, Roche) targeting 97 genes recurrently mutated in myeloid malignancies and clonal hematopoiesis spanning 347 kb (Table S2). Amplified DNA libraries were hybridized to the capture probes in pools of 10-12 samples according to the manufacturer’s instructions. The captured DNA libraries were amplified by 14 cycles of PCR using a KAPA HiFi HotStart DNA polymerase and purified using AMPure XP Beads.

Post-capture amplified DNA libraries were quantified by Qubit (Life Technologies) and size distribution and quality analyzed using a Bioanalyzer chip (Agilent Technologies). Libraries were pooled in equimolar concentrations and were sequenced on an Illumina NextSeq 500 using paired-end reads.

##### Somatic variant calling

Sequencing data were analyzed with a custom Python pipeline based on the GATK Best Practices (GATK v4.1.2.0 and Picard v2.21.3). Raw sequencing reads were converted to an unmapped BAM file and adapter sequences soft-clipped using Picard *MarkIlluminaAdapters*. Following conversion back to a FASTQ file, reads were mapped to the hg38 human reference genome assembly using the Burrows–Wheeler aligner v0.7.17^103^ with alternate contig-aware alignment. Mapped and unmapped BAM files were merged using *MergeBamAlignment* and reads from different sequencing lanes were combined. Duplicate reads were marked using Picard *MarkDuplicates* and base quality scores recalibrated with GATK BaseRecalibrator and ApplyBQSR. Somatic variant calling was performed on the pre-processed BAM files using VarDictJava v1.7.0^104^ and Mutect2^108^ in tumor-only mode. For VarDict, variants were called with a minimum variant allele frequency of 0.01, minimum base quality score of 25 and minimum supporting reads of 2, with indel realignment and removal of adapter sequences. For Mutect2, a minimum tumor LOD of 2 was used and variants were filtered for sequence context-dependent artefacts using *FilterMutectCalls* and *FilterByOrientationBias*. Indels were left-aligned and normalized using bcftools norm (v1.9). Variants were annotated using Annovar.^105^ Target enrichment metrics and coverage was calculated using Picard *CollectHsMetrics* and custom scripts.

VarDict and Mutect2 variant calls were analyzed separately to identify a consensus list of high-confidence variants. The following post-processing filters were applied to VarDict calls to exclude likely sequencing artefacts:

1. Minimum of 5 variant reads for SNVs (with at least 2 reads in forward and reverse directions), or minimum of 10 variant reads for indels (with at least 4 reads in forward and reverse directions).
2. Minimum base quality score 30.
3. Minimum mapping quality score 40, except for variants in *U2AF1;U2AF1L5*, where the mapping quality was ignored. This is because in hg38, there is a duplication of the *U2AF1* gene on chromosome 21 called *U2AF1L5*, which results in reads being flagged as multi-mapped.
4. Maximum strand bias Fisher *p*-value of 0.0001.
5. No position bias towards beginning or end of reads.

The following post-processing filters were applied to Mutect2 calls:

1. Passed all default Mutect2 filters or only failing the *clustered_events* filter.
2. Minimum of 5 variant reads for SNVs (with at least 2 reads in forward and reverse directions), or minimum of 10 variant reads for indels (with at least 4 reads in forward and reverse directions).

Variants were flagged as likely germline, or sequencing artefacts, if any of the following applied:

1. Variant allele with a population allele frequency >1 in 1,000 according to any of three large polymorphism databases (Gnomad, 1000 Genomes Project, ESP6500) that is not a hotspot driver mutation with a COSMIC (v88) occurrence count of >100 cases or is present in a list of clonal hematopoiesis-associated mutations compiled from five large studies.^14^^,^^15^^,^^17^^,^^19^^,^^109^
2. Variant allele frequency (VAF) between 0.4–0.6 or >0.9 unless recurrent in COSMIC >5 times, or previously reported in clonal hematopoiesis.
3. Present in a panel of normal cord blood samples.
4. Recurrent in the cohort unless present at least 5 times in COSMIC or at least 2 times in the clonal hematopoiesis studies.

After filtering, variants were manually inspected using the Integrative Genomics Viewer (IGV) tool (http://software.broadinstitute.org/software/igv/).

##### Annotation of pathogenic driver variants

Samples were annotated as having CH based on the presence of at least one driver mutation in bone marrow sequencing at VAF ≥ 0.01. Variants were annotated as pathogenic driver mutations using the following criteria:

1. Truncating mutations (nonsense, splice site or frameshift indel) in the following genes: *DNMT3A*, *TET2*, *ASXL1*, *NF1*, *IKZF1*, *RAD21*, *WT1*, *KMT2D*, *SH2B3*, *TP53*, *CEBPA*, *RUNX1*, *BCOR*, *KDM6A*, *STAG2*, *PHF6*, *KMT2C*, *KMT2E*, *PPM1D*, *ATRX*, *EZH2*, *CREBBP*, *NOTCH1*, *CUX1* and *ZRSR2*.
2. Non-synonymous variants at the following hotspot residues: *CBL* E366, L380, C384, C396, C404 and R420; *DNMT3A* R882; *FLT3* D835; *IDH1* R132; *IDH2* R140 and R172; *JAK2* V617F; *KIT* W557, V559 and D816; *KRAS* G12, G13, Q61 and A146; *MPL* W515; *NRAS* G12, G13 and Q61; *SF3B1* K666 and K700; *SRSF2* P95; *U2AF1* S34, R156 and Q157.
3. Non-synonymous variants occurring within the following residues of *DNMT3A*: p.292-350, p.482-614 and p.634-912; *TET2*: p.1104-1481 and p.1843-2002; or *NOTCH1*: p.1574-1620, p.1671-1721.
4. Truncating variants in *CALR* exon 9.
5. *FLT3* internal tandem duplications.
6. Non-synonymous variants reported at least 10 times in COSMIC with VAF < 0.4.
7. Non-synonymous variants falling within an annotated InterPro domain with VAF < 0.4.
8. Non-synonymous variants reported in COSMIC > 100 times.

If a variant did not meet these criteria, it was annotated as a variant of unknown significance (VUS).

##### Comparison of bone marrow and peripheral blood allele frequencies

Variants were called in peripheral blood DNA sequencing data as described above, using a minimum VAF cutoff of 0.005. Unfiltered variant calls were intersected with the list of curated bone marrow variants to compare the VAF between bone marrow and blood. For those mutations known to be present in bone marrow which were not called in peripheral blood, raw allele counts were performed directly from the BAM files using bcftools mpileup (with minimum base quality of 30 and minimum mapping quality of 35), and the allele frequency was calculated.

#### Flow cytometry and FACS sorting

Thawing media was prepared with IMDM medium (Gibco) supplemented with 20% FBS and 110 μg/mL DNase. Bone marrow samples were thawed at 37°C in a water bath, 1 mL warm FBS was added, and the suspension then diluted by dropwise addition of 8 mL thawing media. The suspension was centrifuged at 400 × g for 10 mins, cells were resuspended in flow cytometry staining medium (IMDM with 10% FBS and 10 μg/mL DNase), filtered through a 35 μm cell strainer, and placed on ice.

Cells were stained with antibodies listed for 20–30 min on ice. Following antibody incubations, cells were washed with 1 mL flow cytometry staining buffer, centrifuged at 350 × g for 5 min, and resuspended in flow cytometry staining buffer containing the live/dead stain.

For flow cytometry analysis of BM samples, unseparated BM MNCs were used. Samples were stained with the following antibodies: anti-CD38-BV421 (1:20, Biolegend, clone HIT2), anti-CD10-BV605 (1:40, Biolegend, clone HI10a), anti-CD49f-BV650 (1:40, Biolegend, clone GoH3), anti-CD117-BV785 (1:40, Biolegend, clone 104D2), anti-CD45RA-BB515 (1:40, BD, clone HI100), anti-CD123-PE (1:40, Biolegend, clone 6H6), anti-CD90-PE/Cy7 (1:20, Biolegend, clone HI100), anti-CD34-APC (1:160, Biolegend, clone 581), anti-CD2-PE/Cy5 (1:160, Biolegend, clone RPA-2.10), anti-CD3-PE/Cy5 (1:320, Biolegend, clone HIT3a), anti-CD4-PE/Cy5 (1:160, Biolegend, clone RPA-T4), anti-CD8a-PE/Cy5 (1:320, Biolegend, clone RPA-T8), anti-CD11b-PE/Cy5 (1:160, Biolegend, clone ICRF44), anti-CD14-PE/Cy5 (1:160, eBioscience, clone 61D3), anti-CD19-PE/Cy5 (1:160, Biolegend, clone HIB19), anti-CD20-PE/Cy5 (1:160, Biolegend, clone 2H7), anti-CD56-PE/Cy5 (1:80, Biolegend, clone MEM188), anti-CD235ab-PE/Cy5 (1:320, Biolegend, clone HIR2), and propidium iodide (final concentration 3 μM; Biolegend) was used for dead cell exclusion. Analysis was done on a LSR Fortessa X20 (BD Biosciences). Unstained, single-stained (CompBeads, BD Biosciences), and fluorescence-minus-one (FMO) controls were used to determine background staining and compensation in each channel. Gating was kept consistent across all samples to enable quantification of population sizes.

For FACS sorting of BM samples for TARGET-seq+ and ddPCR, either unseparated BM MNCs or CD34-enriched BM MNCs were used. Samples were stained with the following antibodies: anti-CD38-BV421 (1:20, Biolegend, clone HIT2), anti-CD10-BV605 (1:40, Biolegend, clone HI10a), anti-CD117-BV785 (1:40, Biolegend, clone 104D2), anti-CD45RA-BB515 (1:40, BD, clone HI100), anti-CD123-PE (1:40, Biolegend, clone 6H6), anti-CD49f-PE/Dazzle594 (1:160, Biolegend, clone GoH3), anti-CD90-PE/Cy7 (1:20, Biolegend, clone HI100), anti-CD34-APC (1:160, Biolegend, clone 581), anti-CD2-PE/Cy5 (1:160, Biolegend, clone RPA-2.10), anti-CD3-PE/Cy5 (1:320, Biolegend, clone HIT3a), anti-CD4-PE/Cy5 (1:160, Biolegend, clone RPA-T4), anti-CD8a-PE/Cy5 (1:320, Biolegend, clone RPA-T8), anti-CD11b-PE/Cy5 (1:160, Biolegend, clone ICRF44), anti-CD14-PE/Cy5 (1:160, eBioscience, clone 61D3), anti-CD19-PE/Cy5 (1:160, Biolegend, clone HIB19), anti-CD20-PE/Cy5 (1:160, Biolegend, clone 2H7), anti-CD56-PE/Cy5 (1:80, Biolegend, clone MEM188), anti-CD235ab-PE/Cy5 (1:320, Biolegend, clone HIR2), and 7-AAD (Biolegend) was used for dead cell exclusion. For sample NOC156, cells were stained with the same panel except anti-CD49f, and anti-CD117 was substituted for anti-CD117- PE/Dazzle594 (1:80, Biolegend, clone 104D2). Single-cell index sorting was performed on a Sony MA900 into 384-well plates containing 3 μL lysis buffer (except for optimization experiments, which were done in 96-well plates). Unstained, single-stained, and FMO controls were used to determine background staining and compensation in each channel. Doublets and dead cells were excluded. The following populations were sorted: Live/Lin^–^CD34^+^, Live/Lin^–^ CD34^+^CD38^–^, and CD34^–^ cells (except for sample NOC156, where only CD34^+^ cells were analyzed). In addition to the sample of interest, cells from the NOC153 control sample were sorted onto every plate, making up approximately 10% of wells on each plate, and two empty wells were used as no-template controls. After sorting, each plate was centrifuged and snap frozen on dry ice prior to storage at –80°C.

Flow cytometry data analysis was performed using FlowJo v10.8.1 and R.

#### Whole-genome sequencing (WGS) analysis

For validation of clonal cell fractions detected by TARGET-seq+, we analyzed data from our separate study in which WGS was performed on FACS-sorted Lin^–^CD34^+^ cells.^110^ bcftools mpileup was used to calculate the VAF of each mutation from the BAM files, and the mutant cell fraction was calculated as 2 × VAF for autosomal mutations.

#### Droplet digital PCR (ddPCR)

For CH samples NOC002 (2 loci), NOC115 (2 loci), and NOC132 (1 locus), ddPCR on sorted immunophenotypic populations was performed to validate the frequency of mutations screened by TARGET-seq+. For each locus, we first determined the optimal DNA strand for the WT probe to outcompete the binding of the mutant probe to the WT allele, and vice-versa, using the T_m_ mismatch option from the IDT OligoAnalyzer tool. Next, for each locus, primers and probes were designed with Primer3Plus, using the Internal Oligo option. Binding specificity of the primers and of the reference allele probes was checked with Primer BLAST, and the Hetero-Dimer option from the IDT OligoAnalyzer was used to control for primer and probe cross-binding. For all loci, the WT probe contained the HEX fluorophore, and the mutant probe contained the 6-FAM fluorophore at the 5’ end.

Primers for all loci were first tested for specificity and successful amplification in a bulk PCR. Next, the optimal annealing temperature for each primer/probe set was determined by an annealing temperature gradient experiment, using gDNA from the respective samples (extracted either from BM MNCs or PB granulocytes) for the mutant allele probes, or a WT control gDNA (extracted from PB of a WT donor) for the reference allele probes.

BM samples were then thawed and stained with antibodies for FACS sorting, as detailed above. Up to 1000 cells from immunophenotypic populations of interest were sorted on purity mode into tubes containing 5 μL of lysis buffer consisting of 40 mM Tris-HCl pH 8.0 (Thermo Scientific), 0.2% Triton X-100 (Sigma-Aldrich), and 2 x 10^-04^ AU/μL protease (Qiagen). Where possible, multiple replicates (2-3) of the same population were sorted. Cell pellets were incubated at 56°C for 1 h, followed by incubation at 72°C for 15 min to perform lysis and protease heat-inactivation. PCR was set up using 11 μL of 2X ddPCR supermix for probes (no dUTP), 1.1 μL of the 20X primer/probe mix (containing each primer at 18 μM and each probe at 5 μM), 0.22 μL of MseI enzyme, 3-5 μL of cell lysate or gDNA, and 4.7-6.7 μL of nuclease-free water. Droplets were generated using a Bio-Rad Automated Droplet Generator. The following PCR program was used: 95°C for 10 min for initial denaturation, 40 cycles of 94°C for 30 s and 59/60/63°C for 1 min. Final incubation was performed at 98°C for 10 min. Droplets were read with a Bio-Rad QX200 Droplet Reader. No-template controls, WT gDNA negative controls, and sample gDNA positive controls were used to set gates for mutant, WT, and double positive droplets. Fractional abundance (VAF) in each replicate was calculated with the QX Manager Software v2.1.

#### TARGET-seq+ library preparation

##### Primer design

Targeted genotyping primers used in the pre-amplification RT-PCR step were designed to amplify regions 180–900 bp long. Where possible, gDNA primers were designed to anneal within intronic regions flanking the mutation of interest, while mRNA primers were designed to anneal to exonic regions outside of the gDNA amplicon, so that independent amplicons would be generated from mRNA and gDNA. Furthermore, when a heterozygous SNP was observed close to the mutation, primers were placed in order to cover the SNP within the amplicon, enabling a direct measurement of allelic dropout. Primers were designed with Primer3Plus^106^ and specificity was checked using Primer-BLAST.^111^ For each target, primer pairs were tested for specificity and efficiency in bulk PCR reactions and in single cells.

Targeted primers for use in the genotyping PCR1 step were designed to be nested within each of the amplicons generated in the pre-amplification RT-PCR. Nested amplicons were 290–631 bp in length. As for pre-amplification primers, gDNA primers were designed to anneal within intronic regions flanking the mutation, and cDNA primers were designed to anneal to exonic regions outside of the gDNA amplicon, to generate independent mutational readouts from cDNA and gDNA, where possible. Primer pairs were tested for specificity and efficiency in bulk PCR reactions and in single cells.

##### Lysis buffer preparation

Lysis buffer was prepared as described in Table S4, consisting of 0.1% Triton X-100 (Sigma-Aldrich), 0.5 mM dNTPs (Life Technologies), 5% PEG 8000 (Sigma-Aldrich), 0.5 U/μL RNase inhibitor (Takara), 2.7 x 10^-05^ AU/μL protease (Qiagen), and 1:8,000,000 diluted ERCC spike-in mix (Ambion). 25 μL of lysis buffer was dispensed into each well of a 384-well stock plate using a Formulatrix Mantis with a high-volume chip, and 1.79 μL of 10 μM barcoded oligodT-ISPCR primer was added to each well using an INTEGRA Viaflow. 3 μL of barcoded oligodT-lysis buffer mix was then transferred into each well of a 384-well plate. Plates were sealed and stored at –80°C and thawed prior to cell sorting.

##### Reverse transcription and pre-amplification

Plates containing sorted cells were removed from –80°C storage and incubated at 72°C for 15 min to perform cell lysis, RNA denaturation, and protease heat inactivation. For reverse transcription (RT), 1 μL of RT mix was added, bringing reaction concentrations to 25 mM Tris-HCl (Thermo Scientific), 30 mM NaCl (Invitrogen), 2.5 mM MgCl_2_ (Invitrogen), 1 mM GTP (Thermo Scientific), 8 mM Dithiothreitol (DTT, Thermo Scientific), 0.5 U/μl RNase inhibitor (Takara), 2 μM of Smart-seq2 template switching oligo (TSO, IDT), 2 U/μl of Maxima H-minus reverse transcriptase enzyme (Thermo Scientific), and target-specific mRNA primers (70 nM final concentration). RT was performed by incubation at 42°C for 90 min followed by 10 cycles of 50°C for 2 min and 42°C for 2 min. The reaction was terminated by incubating at 85°C for 5 min.

Pre-amplification PCR mix containing target-specific genotyping primers was prepared as described in Table S4, to achieve reaction concentrations of 1× KAPA HiFi HotStart Ready Mix (Roche), 50 nM ISPCR primer, 28 nM target-specific cDNA primers, and 400 nM target-specific gDNA primers. Pre-amplification PCR was performed directly after reverse transcription by addition of 6 μL PCR mix and incubation on a thermocycler using the following program: 98°C for 3 min for initial denaturation, 21 cycles of 98°C for 20 s, 67°C for 30 s and 72°C for 6 min. Final elongation was performed at 72°C for 5 min. Conditions used for all RT-PCR steps are listed in Table S4. The sequences of the primers used in the RT-PCR steps for whole transcriptome amplification and targeted genotyping amplification are listed in Table S4.

Following cDNA amplification, successful libraries contain whole transcriptome cDNA and amplicons spanning each targeted mutation. An aliquot of this cDNA-amplicon mix was used for whole transcriptome library preparation and another aliquot for single-cell genotyping library preparation. 1 μL of cDNA-amplicon mix was pooled per well to create cDNA pools from 192 uniquely barcoded single-cell libraries, using a Mosquito HTS liquid handling platform (TTP Labtech). Each cDNA pool was purified twice using Ampure XP beads with 0.6:1 beads to cDNA ratio. Pooled cDNA libraries were checked using a High Sensitivity DNA Kit on a Bioanalyzer (Agilent) or a High Sensitivity NGS Fragment Analysis Kit (1 bp - 6,000 bp) on a Fragment Analyzer (Agilent). Libraries were quantified by Qubit dsDNA HS Assay (Life Technologies). These pools were used to generate 3’ biased whole-transcriptome libraries. The remainder of the cDNA-amplicon mix was diluted 1:2 with water and stored at –20°C for use in single-cell genotyping.

##### Whole-transcriptome library preparation and sequencing

Bead-purified cDNA pools were used for tagmentation-based library preparation with a Nextera XT DNA Library Preparation Kit (Illumina) using a custom PCR amplification strategy to generate 3′ biased libraries containing oligodT cell barcodes as previously published,^59^ with some modifications. Pooled cDNA libraries were diluted to 800 pg/μL and a total of 4 ng (5 μL) from each pool was used in the tagmentation reaction with 10 μL tagmentation buffer (TD) and 5 μL ATM enzyme. The reaction was incubated at 55°C for 10 min, followed by the addition of 5 μL 0.2% SDS to release Tn5 from the DNA. Library amplification was performed using 5 μL Nextera XT i7 forward index primer (Illumina) and 5 μL custom i5 index primers (2 μM) (see Table S4 for sequences). The custom i5 index primer binds the barcoded oligodT-ISPCR adapter, resulting in amplification of the 3’ fragments containing the cell barcode. PCR was performed by adding NPM enzyme (Nextera XT DNA Library Preparation Kit, Illumina) and incubation on a thermocycler using the following program: 95°C for 30 s, 14 cycles of 95°C for 10 s, 55°C for 30 s and 72°C for 30 s, and then a final elongation of 5 min at 72°C. After tagmentation, each indexed pool was purified twice with Ampure XP beads using a 0.7:1 beads to cDNA ratio. Library quality was checked using a High Sensitivity DNA Kit on a Bioanalyzer and quantified using Qubit dsDNA HS Assay. Equimolar pools were made and sequenced using custom sequencing primers for Read1 and Index2 (P5-SEQ, I5-SEQ, 300 nM in HT1 buffer, see Table S4). For benchmarking experiments, libraries were sequenced on a NextSeq 500/550 High Output v2.5 (75 cycle) kit (Illumina) using the following sequencing configuration: 15 bp R1; 8 bp index read; 69 bp R2. For the main experiments, up to 9,984 single-cell libraries (52 pools of 192 single-cell libraries) were sequenced on a NovaSeq S4 flow cell with a targeted sequencing depth of 1 million reads/cell using the following sequencing configuration: 15 bp R1; 8 bp index read 1; 8 bp index read 2; 200 bp R2.

##### Targeted single-cell genotyping

To generate Illumina-compatible libraries for single-cell genotyping, two PCR steps were performed as previously published in the TARGET-seq protocol.^59^ As the genotyping amplicons generated by the pre-amplification RT-PCR are not barcoded, genotyping PCR reactions were carried out separately for each single-cell library.

In the first PCR step (genotyping PCR1), nested target-specific primers containing universal CS1 (forward primer) or CS2 (reverse primer) adapters are used to amplify the target regions of interest. Incorporation of a barcode sequence specific to each plate into these primers enables libraries from different plates to be pooled subsequently. Primer sequences used for genotyping PCR1 for each sample are listed in Table S4. PCR1 reactions were performed using 3.25 μL of KAPA 2G Robust HS Ready Mix (Sigma-Aldrich), 1.5 μL of diluted cDNA-amplicon mix and 300 nM target-specific primers, in a 6.5 μL reaction.

In the second PCR step (genotyping PCR2), Illumina-compatible adapters containing a 10 bp cell barcode are attached to the genotyping PCR1 product by binding to the CS1/CS2 adapters. PCR2 reactions were performed using FastStart High Fidelity polymerase (Sigma-Aldrich) with 1.0 μL of PCR1 product and 1.2 μL of each barcode primer mix (Access Array Barcode Library for Illumina Sequencers- 384, Single Direction, Fluidigm) in a 6.2 μL reaction.

Indexed amplicons were pooled using a Mosquito HTS liquid handling platform and purified with Ampure XP beads using a 0.8:1 beads to PCR product ratio. Purified pools were quantified using Qubit dsDNA HS Assay and the quality checked using a Tapestation High Sensitivity D1000 kit (Agilent) to ensure the size distribution of amplicons was as expected. Each pool was diluted to a final concentration of 4 nM and further diluted to 10 pM in HT1 buffer prior sequencing. Libraries were sequenced on a NextSeq 500/550 Mid Output v2.5 kit (300 cycle) (Illumina) using 150 bp paired-end reads, with 10 bp for the cell barcode index read and custom sequencing primers (Table S4).

#### TARGET-seq+ validation experiments

Validation experiments comparing TARGET-seq+ with TARGET-seq were performed in 96-well plates using JURKAT cells and primary human CD34^+^ HSPCs. 3’ TARGET-seq libraries were generated according to the published protocol.^59^ Cells were sorted into 4.1 μL lysis buffer, consisting of 0.18% Triton X-100 (Sigma- Aldrich), 1.0 mM dNTP (Life Technologies), 1.0 U/μl RNase inhibitor (Takara), 2.7 × 10^−5^ AU/mL protease (Qiagen), 1.0 μM barcoded oligodT-ISPCR primer. RT was performed using SMARTScribe enzyme, RNase inhibitor, Smart-seq2 TSO (1 μM final concentration) and targeted mRNA primers (700 nM final concentration). PCR pre-amplification was performed using SeqAmp DNA polymerase, ISPCR primers (50 nM final concentration) and targeted gDNA and cDNA primers. TARGET-seq+ RT-PCR was performed as described above using double volumes per well. For both conditions, 20 cycles of amplification were used for JURKAT cells and 24 cycles of amplification for HSPCs. 3’ transcriptome libraries were prepared as for TARGET-seq+ libraries detailed above and were sequenced on a NextSeq 500/550 High Output v2.5 (75 cycle) kit.

#### Targeted single-cell genotyping analysis

##### Pre-processing and mutation calling

Single-cell genotyping reads were pre-processed using the custom TARGET-seq pipeline (https://github.com/albarmeira/TARGET-seq).^59^ Reads were first demultiplexed using the 384 well barcodes introduced via the genotyping PCR2 reaction, followed by demultiplexing based on plate barcodes introduced during genotyping PCR1. This generated separate FASTQ files for each single cell. Reads were aligned to hg38 using STAR version 2.7.3a with default settings and cDNA/gDNA amplicons were separated into different bam files, extracting reads matching the primer sequences used for targeted PCR barcoding. This allowed independent mutational information to be obtained from cDNA and gDNA amplicons. Variant calling was performed with *mpileup* (samtools version 1.1, options: --*minBQ 30*, --*count-orphans*, --*ignore overlaps*) and results were summarized using the custom pipeline.

For indels, reads from cDNA/gDNA amplicons were separated into different FASTQ files using cutadapt to extract reads matching the primer sequences. The number of reads with the wild-type or mutant sequence was counted for each cell using fastq-grep.

Mutational calling in single cells was then performed with custom R scripts, separately for each mutation. Coverage for each cell was calculated as the sum of all reads across the variant locus for that cell. Empty wells routinely displayed zero or very few reads (usually up to 2), indicating no cross-well contamination. A filtering threshold was applied to remove cells where the amplicon was not detected, or where coverage was too low for reliable genotyping. The minimum coverage was 50 reads for gDNA amplicons and 30 reads for cDNA amplicons. In cells with coverage below the threshold, the amplicon was called undetected.

The single-cell variant allele frequency (scVAF) for each cell was calculated with the following formula:

scVAF=variantreads/coverage

In a single cell, there are only two genomic DNA alleles. In the first cycles of PCR, if the amplification of one allele is favored over the other, there will be unequal amplification of the two alleles. Therefore, it is universal in single-cell genotyping to observe a distribution of scVAFs that will range from ∼1% (0.01) to ∼99% (0.99).^59^^,^^99^^,^^112^ This scVAF distribution is also observed for genotyping of mRNA/cDNA. Furthermore, there is an inherent error rate of the DNA polymerase in PCR amplification and in next-generation sequencing, which needs to be accounted for when calling cell genotypes. To define the appropriate scVAF thresholds for CH mutation calling, we adopted a method used in our two prior studies.^99^^,^^112^ Single cells from a control bone marrow sample that was WT for all mutations of interest were FACS-sorted onto every plate and processed in parallel with other wells containing single cells from a CH sample. Since the genotype of these control cells was known *a priori*, these data were used to determine the ‘noise’ or error rate at each locus. We thus used the scVAF distribution from these WT control cells (scVAF_WT-CTRL_) to set thresholds for assigning a genotype to each single cell from samples of interest (Figure S3A). We define two thresholds for calling CH mutations. The lower scVAF threshold for calling a cell WT was set using the following formula:

$$scVAF\;threshold\;for\;calling\;a\;cell\;WT=mean\left(scVAF_{WT-CTRL}\right)+3\times SD(scVAF_{WT-CTRL})$$

Cells with scVAF below this threshold were called WT for that mutation. The scVAF threshold for calling a cell mutant was set using the following formula:

$$scVAF\;threshold\;for\;calling\;a\;cell\;mutant=mean(scVAF_{WT-CTRL})+3\times SD(scVAF_{WT-CTRL})+0.01$$

Cells with scVAF above this threshold were called mutant for that mutation. For example, for the *TET2* pR1261C gDNA amplicon, the scVAF thresholds were calculated as follows:

$$mean(scVAF_{WT-CTRL}):\;0.000719$$

$$SD(scVAF_{WT-CTRL}):\;\;\;0.000472$$

scVAFthresholdforcallingacellWT=0.000719+3×0.000472=0.002136

scVAFthresholdforcallingacellmutant=0.002136+0.01=0.012136

We validated this strategy for setting thresholds by bootstrap resampling of the WT control cell scVAFs and fitting each sample to a beta distribution. The resulting probability density distribution quantifies the probability that the scVAF of a WT control cell falls within a particular range of values. The probability of WT control cells having a scVAF measurement above the threshold used to call cells mutant (i.e. mean + 3^∗^SD + 0.01) ranged from 0 to 0.006. Thus, the likelihood of calling false positives (i.e. incorrectly calling a WT cell mutant) is low with this strategy.

Furthermore, we required a minimum number of 10 mutant reads for a cell to be called mutant. Cells with a borderline scVAF (between the WT and mutant scVAF thresholds), where the number of mutant reads was < 10, or where allelic dropout (see next section) was confirmed by analysis of a germline SNP, were called “undetermined” and were excluded from downstream analyses comparing genotypes, due to the uncertainty regarding their genotype.

The above strategy was performed separately for gDNA and cDNA amplicons. For each locus, genotyping information from gDNA and cDNA amplicons were then combined, and a consensus genotype assigned. Consensus genotypes were assigned as follows:

1. If the mutation was identified in either the gDNA or cDNA amplicon, the cell was called “mutant”.
2. If both amplicons were WT, the cell was called “WT”.
3. If the gDNA amplicon was WT but the cDNA amplicon was undetected or undetermined, the cell was called “WT”.
4. If the cDNA amplicon was WT but the gDNA amplicon was undetected or undetermined, the cell was called “undetermined”, due to the high allelic dropout rate of cDNA amplicons.

##### Allelic dropout (ADO) detection using germline heterozygous SNPs

Cells that appear WT for a CH mutation can either be genuinely WT (where both alleles have been sampled) or there may have been allelic dropout (ADO) of the allele harboring the mutation (where only the WT allele has been sampled). Germline single nucleotide polymorphisms (SNP) were present within the gDNA amplicon for four CH mutations that we analyzed. These SNPs were all heterozygous in the CH samples, but homozygous in the WT-control sample. Since both alleles are present in every cell, these SNPs can be used to determine whether both alleles have been sampled in cells from CH samples, or whether ADO has occurred.

We calculated the scVAF of the SNP with the following formula:

$$SNP\;scVAF=reads\;with\;alternate\;allele\;/\;\left(reads\;with\;alternate\;allele+reads\;with\;reference\;allele\right)$$

We defined the allele observed in the WT control sample as the “reference” (Ref) allele for each SNP, whereas the allele observed only in the CH sample was the “alternate” (Alt) allele. Thus, WT control cells were homozygous Ref (SNP scVAF close to 0), whereas CH sample cells could be heterozygous, homozygous Ref (where there was ADO of the Alt allele) or homozygous Alt (where there was ADO of the Ref allele).

First, we established whether the SNP Alt allele and the CH mutation were found on the same allele (i.e. they were in-phase), or on opposite alleles (i.e. they were out-of-phase), by examining the correlation between SNP and CH mutation scVAFs (Figures S3E and S3F). A positive correlation indicated the SNP Alt allele and the CH mutation were in-phase, while a negative correlation indicated they were out-of-phase.

Next, we called SNP genotypes for each single cell. scVAF thresholds for calling SNP genotypes were determined in a similar way as for CH mutation calling, based on the SNP scVAF distribution in cells from the WT control sample (Figure S3G). Since the control sample was homozygous reference (Hom Ref) for each SNP, this enabled the error rate at each locus to be determined. The SNP scVAF threshold for detection of the SNP Alt allele was calculated as follows:

$$scVAF\;threshold\;for\;detection\;of\;the\;SNP\;Alt\;allele=mean\left(scVAF_{WT-CTRL}\right)+3\times SD(scVAF_{WT-CTRL})+0.01$$

Cells with SNP scVAF below this threshold were called Hom Ref for the SNP (i.e. ADO of the SNP Alt allele had occurred).

The SNP scVAF threshold above which a cell was called homozygous alternate (Hom Alt) for the SNP was calculated as the inverse of the lower threshold:

SNP Hom Alt scVAFthreshold=1-scVAFthresholdfordetectionoftheSNPalternateallele

Cells with scVAF above this threshold are called Hom Alt (i.e. ADO of the SNP Ref allele had occurred), while cells with VAF between the two thresholds were called heterozygous (Het; i.e. biallelic detection).

We then used the SNP genotype calls and the phasing information to establish whether the allele harboring the CH mutation was detected:

- When a cell was called “mutant” for the CH mutation, the SNP analysis is not required.
- When the SNP Alt allele and the CH mutation were in-phase, cells that appeared WT for the CH mutation, and Hom Ref for the SNP were called “undetermined” (because ADO of the mutant allele had occurred), whereas cells that were Het or Hom Alt for the SNP were called “WT” for the CH mutation (because the allele containing the CH mutation was detected).
- When the SNP Alt allele and the CH mutation were out-of-phase, cells that appeared WT for the CH mutation, and Hom Alt for the SNP were called “undetermined” (because ADO of the mutant allele had occurred), whereas cells that were Het or Hom Ref for the SNP were called “WT” for the CH mutation, mutation (because the allele containing the CH mutation was detected).

Undetermined cells were excluded from downstream analyses between genotypes (given that the allele containing the mutation was not captured due to ADO).

For each allele, we then calculated the fraction of cells in which ADO occurred, by dividing the number of cells in which ADO of one allele occurred by the total number of sampled cells which passed genotyping quality control (Figure S3H).

We note that ADO of the WT allele has no impact on downstream analysis. As all the CH mutations genotyped in this study are heterozygous (apart from the hemizygous *BCORL1* locus), cells in which ADO of the WT allele occurred (which leads to a heterozygous mutant cell being called homozygous mutant) were retained for downstream analyses. We do not distinguish between heterozygous and homozygous mutant calls for CH mutations in downstream analyses comparing genotypes.

##### ADO estimation using homozygous mutant frequencies

For the loci which did not contain a germline heterozygous SNP in the gDNA amplicon, a direct measure of ADO was not possible. If CH mutations at these loci are assumed to be heterozygous, homozygous mutation calls arise when there is ADO of the WT allele. The WT ADO rate can therefore be estimated by quantifying the fraction of mutant cells that are called homozygous mutant. To identify homozygous mutant cells, we used the inverse of the scVAF threshold used for calling mutant cells (Figure S3I):

scVAFthresholdforcallingacellhomozygousmutant=1-scVAFthresholdforcallingacellmutant

Next, we calculated the fraction of cells in which we inferred ADO of the WT allele occurred, with the following formula:

$$WT\;ADO\;rate=n^{o}\;homozygous\;mutant\;cells\;/\;\left(n^{o}\;heterozygous\;mutant\;cells+2\times n^{o}\;homozygous\;mutant\;cells\right)$$

We first validated this strategy for the 4 loci in which the germline SNP was also present, by comparing WT ADO rates estimated in this way with those calculated with the SNP analysis. Importantly, ADO rates were comparable between the two strategies. Notably, our analysis of germline SNPs also showed that ADO rates for each allele are similar at a given locus (i.e. WT ADO and mutant ADO rates for a single locus are similar; Figure S3H). Hence, the WT ADO rate is a reasonable estimate of the ADO rate for the mutant allele.

For mutations where both gDNA and cDNA genotyping amplicons were used, the WT ADO rate was calculated based on the frequency of cells that were homozygous mutant in both gDNA and cDNA amplicons readouts. Estimated ADO percentages at each locus using this strategy are shown in Figure S3J.

##### Alternative mutation calling strategy using the minima of the VAF distribution

An alternative strategy for setting thresholds for calling mutant cells based on the scVAF distribution obtained for heterozygous germline SNPs. Plotting the distribution of the scVAFs at a given locus across all genotyped cells results in a histogram containing three peaks: one centered at scVAF ∼0.5 and two at values closer to 0 and 1. There will be two minima in the distribution between the three peaks, which can be interpreted as the point that maximally separates heterozygous cells from homozygous cells. In our data, these minima are located at scVAFs equal to ∼0.15 and ∼0.85. We benchmarked this strategy in our dataset.

Cells with scVAFs between the two minima are likely to be heterozygous, whereas the genotype of cells with scVAFs outside this range is less certain. There are two possible mechanisms by which cells may have VAFs at the extremes of the distribution; either: a) one allele was not amplified (i.e. ADO) and this represents noise in the sequencing data (e.g. PCR error during library preparation or sequencing), leading to a VAF measurement that is not equal to 0 or 1; or b) both alleles were amplified but there was considerable allelic bias/skewing during PCR amplification.

If the first mechanism was the dominant effect, one would expect cells that are truly homozygous to show a similar distribution of scVAFs in the range between 0 and ∼0.15. However, the distribution of scVAFs observed in the homozygous reference WT control sample is narrower for all 4 SNPs, suggesting this is not the case. Extrapolating these data to heterozygous CH mutations suggests that most cells with scVAFs in the range between the upper bound of the WT control reference range (∼0.01) and ∼0.15 are likely to be heterozygous mutant with biallelic detection.

Nonetheless, the genotype of cells with scVAFs in the range between the upper bound of the WT control reference range and ∼0.15 is less certain than for cells with scVAFs > 0.15, and we cannot exclude that there was ADO in some cells within this scVAF range. If thresholds for calling heterozygous detection are set based on the minima of the scVAF distribution, the mean ADO rate for the four SNPs is 14.6% per allele, compared to 9.4% using the thresholds defined by the WT control VAF distribution.

To further determine whether our conclusions hold if the analysis is limited to only the highest-confidence mutant cells, we re-called genotypes based on a scVAF threshold of 0.15. Using the 0.15 threshold to call mutant cells, a total of 183 cells originally called CH^MUT^ were reassigned as “Undetermined” (5.8% of all CH-mutant cells). We repeated the transcriptomic analyses comparing CH^MUT^ and CH^WT^ HSC/MPPs with the new genotyping calls. We observed similar results as in our original analysis, albeit with reduction in enrichment scores and level of significance (Table S6). This suggests our genotyping calling strategy using WT control cells to set thresholds better distinguishes between genotypes and leads to a lower false negative rate (fewer mutant cells being assigned as “Undetermined”).

##### Inference of clonal hierarchies

In samples with multiple mutations, the pattern of mutational co-occurrence was used to determine clonal structures and assign a clonal identity to each cell as previously described.^99^^,^^112^ In samples where mutations were mutually exclusive, such as in samples NOC131, NOC117, and NOC115, it was clear that these belonged to independent clones. In cases where mutations co-occur in the same cells, a linear or branching clonal structure may be present. We used infSCITE^113^ to determine the phylogenetic tree which represented the statistically most likely course of somatic events. As input, we used the matrix containing the mutational status for each locus in each cell and ran infSCITE with default parameters and ‘-r 200 -L 10000 -fd 0.01 -ad 0.02 -e 0.2 -p 1000’. We confirmed each phylogenetic tree was consistent with the frequency of cells of each genotype and the clonal size determined by bulk BM sequencing VAF.

The occurrence of ADO means that in some cells, a mutation that is present will not be detected. In some of these cases, we were still able to assign a cell to a clone. For example, in sample NOC002 there were 12 cells in which the *TET2* p.R1261C mutation was detected, but the ancestral *TET2* p.Q726X mutation was not detected. In these cases, we inferred that ADO of the ancestral mutation had occurred, and the cell was assigned to the appropriate daughter clone. In all cases, this was a rare occurrence, consistent with our estimates of the ADO rate.

For all downstream analyses, including differential gene expression, the clone assignment rather than the raw genotype was used to categorize WT and mutant cells.

#### Analysis of FACS index data

Flow cytometry index data were recorded for each single cell during FACS sorting for TARGET-seq+. Fluorescence values were recorded for forward scatter (FSC), back-scatter (BSC; equivalent to side scatter, SSC), Lineage/live/dead, CD34, CD38, CD117, CD45RA, CD10, CD90, CD123, and CD49f (except for the NOC156 control sample). Index data were matched with single-cell identifiers based on the well coordinate and combined with genotyping calls and other metadata into a unified data set. Virtual FACS gating was performed in R based on the sorting strategy. Gates were set based on populations that were negative for each marker. Cells were labelled as positive or negative for each surface marker, and Boolean logic used to assign an immunophenotypic population label. For example, cells that were Lin^–^CD34^+^CD38^–^CD10^–^CD45RA^–^CD90^+^ were labelled as immunophenotypic HSC.

#### Single-cell transcriptome data pre-processing

##### Mapping and transcript counting

Transcriptome sequencing data were demultiplexed into FASTQ files for each plate with a unique i7-i5 index combination using bcl2fastq. These files contained reads from up to 384 cells with shared plate indexes. A custom python pipeline was used to further demultiplex and map the sequencing reads. First, reads from each plate were demultiplexed using the 14 bp single-cell barcode sequence in Read1 using cutadapt (v3.4). Concurrently, cDNA reads (Read2) were trimmed for polyA tails, Nextera adapters and low-quality reads. This generated individual FASTQ files with single-ended cDNA reads corresponding to each single-cell barcode. Reads were then mapped to the hg38 reference genome and ERCC92 transcripts with STARsolo (v2.7.10a) using the GENCODE v38 reference gene annotation (filtered to include protein coding genes and long non-coding RNAs), and counts for each gene were obtained using default parameters except the following: ‘--soloType SmartSeq --soloFeatures GeneFull_Ex50pAS’. Sequencing and mapping quality metrics were calculated with FastQC (v0.11.9), Samtools flagstat (v1.12), MultiQC (v1.11) and the outputs of STAR.

##### Transcript detection and dropout frequency calculation

For the comparison of transcript detection sensitivity between TARGET-seq and TARGET-seq+ (Figures S1G and S1H), data were first downsampled to 5 × 10^5^ reads per cell to remove differences due to unequal sequencing depth. The number of genes detected per cell was calculated as the sum of genes with at least one assigned read.

For calculation of dropout rates (Figure S1I), data downsampled to 5 × 10^5^ reads per cell were used. A random sample of 16 cells per chemistry were compared for JURKAT and 20 cells per chemistry for HSPC. The dropout frequency for a given gene was calculated as the percentage of cells in which the gene was not detected (normalized counts < 1). Genes were divided into three groups to compare the dropout rate in: a) all expressed genes, defined as genes detected in at least 2 cells by any method; b) frequently expressed genes, defined as genes detected in > 50% of all cells; and c) lowly expressed genes, defined as genes with a mean of 2–10 normalized counts per cell.

##### Cell-to-cell correlation analysis

Cell-to-cell correlations for JURKAT cells processed with each method (Figure S1J) were calculated using pairwise Pearson correlations in libraries downsampled to 5 × 10^5^ reads per cell.

#### Single-cell transcriptome analysis

##### Quality control, normalization, and variable gene identification

Single-cell transcriptome analysis was performed using the SingCellaR package (v1.2.1, https://github.com/supatt-lab/SingCellaR).^65^ Metadata including genotyping and FACS index data were matched with single-cell identifiers based on the plate and well coordinates. Cells meeting the following filtering criteria were included in the analysis: reads assigned to genes > 25,000; genes detected > 2,000 and < 15,000; reads assigned to ERCC transcripts < 50%; reads in mitochondrial genes < 15%. Genes expressed in fewer than 10 cells were removed. Reads were normalized by library size using the pool normalization method with prior clustering from the scran package.^114^

##### Dimensionality reduction, data integration and clustering

Variable genes were identified by fitting a generalized linear model to the relationship between the mean expression and squared coefficient of variation (CV^2^) for the ERCC spike-ins, used to estimate technical noise (using the BrenneckeGetVariableGenes function from the M3Drop package).^115^^,^^116^ Genes for which the CV^2^ exceeded technical noise (FDR < 0.05) were considered variable, excluding mitochondrial and ribosomal genes and ERCC transcripts. This identified 17,324 variable genes which were used for principal components analysis (PCA).

Data integration was performed using Harmony^117^ to correct for sample effects, using the sample identifier as the batch, and the top 100 principal components (PCs). The top 100 Harmony-adjusted PCs were then used for Uniform Manifold Approximation and Projection (UMAP) analysis and Louvain graph-based clustering implemented in SingCellaR, with k-nearest neighbors (KNN) equal to 15. Effectiveness of the integration was confirmed by: (a) UMAP visualization pre- and post-integration, to confirm representation of all samples across cell types (Figures S2A and S4A–S4H); (b) comparison of cluster identities across samples, to confirm representation of all samples across clusters, while allowing for unequal distributions between samples as is expected from biological variation; (c) concordance between the cluster assignment of cells and their immunophenotype across samples. For example, we confirmed that the majority of cells in the HSC/MPP cluster consisted of immunophenotypic HSCs and MPPs in all samples, and conversely, the majority of immunophenotypic HSCs and MPPs were assigned to the HSC/MPP cluster.

Clusters were manually annotated based on gene set enrichment of published signatures, immunophenotypic surface marker expression, and expression of canonical marker genes. The SingCellaR ‘identifyGSEAPrerankedGenes’ function was used to pre-rank genes obtained from differential gene expression analysis comparing each individual cluster with all other clusters, and gene set enrichment analysis (GSEA) was performed using the fgsea package (v1.20.0) against gene sets obtained from 9 studies that have characterized human hematopoiesis.^61^^,^^62^^,^^63^^,^^64^^,^^65^^,^^66^^,^^67^^,^^68^^,^^69^ Marker genes differentially expressed in each cluster were identified with the SingCellaR ‘findMarkerGenes’ function, which uses a non-parametric Wilcoxon test on log-transformed, normalized counts, to compare expression levels, and Fisher’s exact test to compare the frequency of cells expressing each gene. Louvain clustering identified 28 clusters, which were collapsed into 23 main clusters based on similarity of GSEA results, marker gene expression and immunophenotype.

The HSC/MPP, LMPP, LMPP cycling, and EMPP clusters were further subclustered using the self-assembling manifolds (SAM) algorithm, using default settings with Harmony-adjusted PCs as input and using the sample identifier as the batch.^90^ The resulting SAM-weighted PCA was then used as input to generate the UMAP in Figure 6A and for Louvain clustering, which identified 7 clusters. For consistency with earlier analyses, these SAM-derived cluster assignments for LMPP, LMPP cycling, and EMPP were used throughout the paper, while cells assigned to the HSC1-3 and MPP clusters were labelled HSC/MPP in Figures 2, 3, 4, and 5.

##### Differential abundance analysis

Differential abundance between sample types (CH vs non-CH) and between mutant and WT cells within CH samples was analyzed using MELD,^118^ a single-cell compositional analysis method that quantifies the likelihood of a cellular state appearing in each sample or condition, and using the Bayesian model scCODA^76^ to determine statistical significance.

For the comparison between sample types (Figures 2F and 2G), only cells sorted as part of the total Lin^–^CD34^+^ gate were included (excluding the Lin^–^CD34^+^CD38^–^ and CD34^–^ sorting strategies), to avoid bias introduced by enrichment of CD38^–^ cells. Sample-associated densities were calculated by running MELD using the Harmony-adjusted PCs as input, and with optimal knn and beta values identified using the MELD parameter search. The mean relative density was calculated using the following formula:

Meanrelativedensity=mean(DensityofCHsamples)/mean(Densityofnon-CHsamples)

Thus, a mean relative density ≥ 1 indicates that the probability of observing a given cell is greater in CH samples compared to non-CH samples, whereas a relative density ≤ 1 indicates that the probability is lower in CH samples compared to non-CH samples. scCODA was run sequentially using all cell types as a reference. No credible differences in abundance between CH and non-CH samples were identified with FDR < 0.2 using any cell type as a reference.

To compare mutant and WT cells within CH samples (Figures 3 and 6), sample and genotype-associated densities were calculated for every genotype by running MELD using the Harmony-adjusted PCs as input, with optimal knn and beta values identified using the MELD parameter search. The relative density of single-mutant and WT cells was then calculated (using L1 normalization to enforce cell-wise sum to be 1), and normalized to the mean relative density in the HSC/MPP cluster for each sample, in order to quantify the relative expansion or contraction of the clone downstream of the HSC/MPP, using the following formula:

Normalizedlikelihood=RelativelikelihoodofMUT:WT/mean(RelativelikelihoodofMUT:WTinHSC/MPP)

These values are visualized per sample in Figures 3D, 3G, 3J, 3N, S4A–S4H, and S6Q. Finally, the normalized likelihood was averaged across samples for visualization in Figures 3C, 3F, and 6G. For the analyses in Figures 3 and S4, scCODA was run using HSC/MPP as the reference cell type. For the analyses in Figure 6, scCODA was run using HSC1 as the reference cell type. Changes classified by scCODA as credible after correction for multiple comparisons (FDR < 0.1 and/or FDR < 0.2) were considered statistically significant.

##### Pseudotime analysis

Diffusion map embeddings^119^ were calculated in scanpy using the Harmony-adjusted PCs as input to the neighborhood graph, excluding the T cell, plasma cell, and endothelial cell clusters. Diffusion pseudotime was then calculated using the HSC at the extreme of the second diffusion component as the root cell. Pseudotime scores were extracted for cells in the HSC/MPP, LMPP, GMP, pDC, and Monocyte clusters and plotted on the UMAP embedding to visualize the myeloid trajectory. For comparison of *TET2*^MUT^ and *TET2*^WT^ cell density along the myeloid trajectory, cells were downsampled to an equal number per sample (n = 176 cells from each of the 4 samples).

##### Differential gene expression analysis

Differential expression testing was performed with a linear mixed model to account for sample covariance using the dream pipeline from the variancePartition package,^120^ which is based on limma-voom.^121^ Testing was performed on uncorrected, raw counts, using the scran normalization size factors. Genes were filtered to exclude genes expressed in fewer than 10% of cells in either group, except for pDCs, monocytes, and Pro-B/Pre-B cells, where a 20% filter was used. A linear mixed model was fitted to each gene using ‘dream’ and differential expression testing was performed using ‘variancePartition::eBayes’. For comparisons between sample types, the sample type was used as the test variable, and the sample identifier, age, sex, and batch effects included as covariates. For comparisons between genotypes within CH samples, the clone was used as the test variable, and the sample identifier and batch effect included as mixed effect covariates. Samples were excluded from the comparison if they had less than 5 cells in either genotype, except for pDCs and monocytes, where a minimum of 2 cells was used. P values were adjusted for multiple testing with Benjamini-Hochberg correction, and differentially expressed genes were defined as those with FDR < 0.1. Analyses were corroborated using a pseudo-bulk approach with EdgeR (results not shown). For defining the CH^WT^ HSC/MPP and non-CH HSC/MPP signatures, thresholds of FDR < 0.1 and log_2_FC > 0.5 were used. Differential expression results are listed in Table S6.

For additional validation, differential expression analysis was performed between CH^MUT^ and CH^WT^ cells within individual CH samples using WilcoxAUC. Testing was performed on scran normalized counts, which were filtered to exclude genes expressed in fewer than 20% of cells in either group.

To assess the impact of ADO rates on differential expression analysis between CH^MUT^ and CH^WT^ cells, a random 10% sample of CH^MUT^ HSC/MPPs was deliberately re-classified as CH^WT^, and the differential expression analysis repeated. GSEA was performed on the resulting ranked gene lists and the results summarized in Table S6.

##### Changes in gene expression along pseudotime

For plotting gene expression along pseudotime, log_2_ normalized expression data were fitted to the pseudotime rank using a generalized additive model (GAM) separately for WT and mutant cells.

##### Gene set enrichment analysis (GSEA)

Gene rankings for gene set enrichment analysis (GSEA) across multiple samples were generated by differential gene expression testing using the dream mixed model, as described above. Genes were ranked by the z statistic from dream. For analyses between CH^MUT^ and CH^WT^ cells within individual CH samples, the gene rankings from wilcoxAUC were used, ranking on the AUC value. To perform GSEA, the fgseaMultilevel function from the fgsea package^122^ was used. Gene sets were obtained from MsigDB v7.5.1 and published studies. Hematopoietic gene sets used in GSEA and AUCell analyses relating to Figures 4, 5, and 6 are listed in Table S5. Significantly enriched gene sets were filtered using the FDR as described in each figure.

##### AUCell signature analysis

The AUCell package (v1.18.1)^82^ was used to quantify the gene set activity in single cells. AUCell gene-expression rankings were created using the SingCellaR ‘Build_AUCell_Rankings’ function. AUCell gene signature enrichment was then calculated using the ‘Run_AUCell’ function with the gene matrix transposed (GMT) file of gene sets. Hematopoietic gene sets were the same as those used for GSEA analysis described above. Differences in mean AUCell scores between WT and mutant cells were tested by a linear mixed model, using clone identity as the fixed effect and sample identity as mixed effects. P-values were obtained by a likelihood ratio test of the full model with the clone effect against the model without the clone effect.

##### SCENIC transcription factor regulon analysis

To infer transcription factor (TF) regulon activity, regulon analysis was performed using pySCENIC.^82^ pySCENIC was run as per the workflow guidelines from Van de Sande et al.^101^ to identify candidate TF-regulons, using the filtered, pre-processed raw counts as the input, and a list of human TFs from Lambert et al.^123^ Candidate regulons were pruned using the annotations of TF motifs ‘motifs-v10nr_clust-nr.hgnc-m0.001-o0.0.tbl’, and CisTarget was applied using the ‘mc_v10_clust’ databases of known human TF motifs annotated at: a) 500 bp upstream and 100 bp downstream of the transcription start site (TSS); and b) 10 kilobases centered around the TSS. No drop-out masking was applied. Enrichment of refined TF regulons was quantified using AUCell, with default parameters. Tests for differential regulon activity were performed using a linear mixed model, as described above. Additionally, WilcoxAUC was used to test differential regulon activity between mutant and WT cells within individual samples. The mean area under the curve (AUC) was calculated to quantify the mean change in activity across samples.

##### Analysis of TCL1A expression

A potential way to assess genotyping accuracy would be to quantify expression of a gene specific for a certain genotype. It has been proposed that *TCL1A* is aberrantly expressed in *TET2*^MUT^ HSCs, and that this effect is reduced by the presence of the rs2887399 variant T allele.^77^ We therefore analyzed the frequency of immunophenotypic HSC/MPPs expressing *TCL1A* transcripts across CH and non-CH samples. *TCL1A*^hi^ cells were defined as cells with > 2 reads mapping to *TCL1A*.

If *TCL1A* expression was a specific marker for *TET2*^MUT^ HSC/MPPs, it would be expected that *TCL1A*^hi^ HSC/MPPs are transcriptionally more similar to *TET2*^MUT^ HSC/MPPs than to other *TET2*^WT^ HSC/MPPs from the same sample. We therefore generated *TET2*^MUT^ and *TET2*^WT^ gene signatures by differential expression analysis between *TET2*^MUT^ and *TET2*^WT^ HSC/MPPs from *TET2*^MUT^ CH samples and validated these by AUCell scoring and ROC AUC analysis with WilcoxAUC (Figures S3M–S3O). We then compared the expression of these signatures in *TCL1A*^hi^ *TET2*^WT^ cells against other *TET2*^WT^ cells and *TET2*^MUT^ cells.

#### FACS sorting and snRNA-seq for ‘in-house’ aging dataset

BM samples were thawed via slow dropwise addition of X-VIVO 10 media (LONZA) with 50% FBS and 100 μg/mL DNaseI (Roche). Cells were centrifuged at 400 × g for 10 min, then dead cell depleted using a commercial kit (EasySep Dead Cell Removal (Annexin V) Kit, STEMCELL) per the manufacturer’s instructions. Cells were resuspended in PBS + 5% FBS and stained for 15 min at RT for fluorescence-activated cell sorting with the following antibodies: anti-CD45RA-FITC (1:50, BD, clone HI100), anti-CD90-PE (1:50, BD, clone 5E10), anti-CD19-BV711 (1:50, BD, clone SJ25C1), anti-CD49f-PE-Cy5 (1:50, BD, clone GoH3), anti-CD271-APC (1:100, Miltenyi, ME20.4-1.H4), anti-CD34-APC-Cy7 (1:200, BD, clone 581), anti-CD38-PE-Cy7 (1:200, BD, clone HB7), anti-CD10-AlexaFluor700 (1:50, BD, clone HI10a), anti-CD14-BV605 (1:200, BD, clone M5E2), anti-CD45-V500 (1:50, BD, clone HI30) and anti-CD33-BV421 (1:100, BioLegend, clone WM53). Cells were washed following staining and resuspended in PBS + 2% FBS containing propidium iodide and filtered through 40μm nylon mesh for cell sorting. Lin^–^CD34^+^CD38^–^ and Lin^–^CD34^+^CD38^+^ populations were sorted into PBS + 0.04% BSA + EDTA on a BD FACSAria Fusion or BD FACSAria III. Cells were counted and Lin^–^CD34^+^CD38^–^ and Lin^–^CD34^+^CD38^+^ cells mixed in the following manner (1:0.33 for 24yM, 1:1 for 26yF, 1:1 for 70yF, and 1:0.5 for 77yF) for downstream 10x Genomics multiome sample preparation by the Princess Margaret Genome Centre.

#### Single-nucleus RNA-seq processing - In-house aging dataset

Single-nucleus RNA-seq processing was performed using Seurat 4.3.0 in R and applied to each sample before merging. The UMI count matrix (BM24M, BM26F, BM70F, and BM77F) was loaded in the R environment using Read10X. Doublets were identified using scDblFinder on the RNA with basic parameters after filtering genes expressed in more than 3 cells, cells with more than 200 features and more than 0.05 percent ribosomal genes. After removing doublets, cells that passed the following filtering criteria were used for downstream analysis: 200 < unique feature counts < 3000, percent mitochondrial genes < 10%. The 10x count matrix for each sample was corrected for ambient RNA contamination using SoupX and used for downstream analysis with the cells that passed quality control. The samples were merged, and the counts were normalized using Scran. The 2000 highly variable features were selected using the “vst” selection method with FindVariableFeatures in Seurat. The cells were scaled, and the samples were integrated using Harmony correcting the sample assignments as a covariate. The optimal number of Harmony-corrected PCA components for downstream analysis was assessed using an elbow plot optimizing at 10. A k-nearest neighbors graph was constructed using FindNeighbors with the Harmony corrected principal components (PCA), and clusters were identified using the Louvain algorithm (resolution = 0.8). A UMAP was constructed using the RunUMAP function at 30 neighbours and 10 Harmony corrected PCA components.

#### Single-cell RNA-seq processing - Ainciburu et al. dataset

The single-cell RNA-seq data was downloaded from GSE18029886, processed using Seurat 4.3.0 in R, and applied to each sample before merging. The UMI count matrix (young1, young2, young3, young4, young5, elderly1, elderly2, elderly3) was loaded in the R environment using Read10X. Doublets were identified using scDblFinder on the RNA after filtering genes expressed in more than 3 cells, cells with more than 200 features and more than 0.05 percent ribosomal genes. After removing doublets, cells passing unique feature count (nFeature_RNA) and percent mitochondrial gene thresholds (percent.mt) were used for downstream analysis (young1: 200 < nFeature_RNA < 4000, percent.mt < 10; young2: 200 < nFeature_RNA < 2700, percent.mt < 10; young3: 200 < nFeature_RNA < 4000, percent.mt < 5; young4: 200 < nFeature_RNA < 4000, percent.mt < 5; young5: 200 < nFeature_RNA < 5000, percent.mt < 10; elderly1: 200 < nFeature_RNA < 4000, percent.mt < 10; elderly2: 200 < nFeature_RNA < 4000, percent.mt < 10; elderly3: 200 < nFeature_RNA < 5000, percent.mt < 10). The samples were merged and normalized using Scran. The 2000 highly variable features were selected using the “vst” selection method with FindVariableFeatures in Seurat. The cells were scaled, and the samples were integrated using Harmony correcting the sample assignments and technology (10x 3’ V2 chemistry, 10x 3’ V3 chemistry) as covariates. The optimal number of Harmony-corrected PCA components for downstream analysis was assessed using an elbow plot optimizing at 15. A k-nearest neighbors graph was constructed using FindNeighbors with the Harmony reduction, and clusters were identified using the Louvain algorithm (resolution = 0.5). A UMAP was generated using the RunUMAP function at 30 neighbours and 15 PCA components.

#### Single-cell RNA-seq processing - Aksöz et al. dataset

This dataset consists of 10x 3’ V2 single-cell RNA-seq data from FACS-purified Lin^–^CD34^+^CD38^–^CD90^+^CD45RA^–^ HSCs from 3 young and 3 aged donors (all male).^85^ Briefly, the raw FASTQ files were aligned against the GRCh38 (Ensembl 93) reference genome (10X Cell Ranger reference GRCh38 v3.1.0) and quantified using the Cell Ranger pipeline (v3.1.0) with default parameters and further processed using Seurat (v4.3.0). Quality control was performed separately for each donor by first filtering out cells with < 200 genes detected, and then retaining only cells with < 10% mitochondrial reads and gene counts that are less than double the median gene count detected in the data for that donor. Genes detected in less than 3 cells were removed. All cells that passed quality control were included in differential expression analysis.

#### Aged vs Young HSC Differential Expression

Pseudobulk profiles of HSCs from each donor were created by taking the sum of all counts for each gene across cells belonging to the HSC cluster within that donor. For the in-house aging dataset, raw counts from young and aged HSC pseudobulks were modeled with DESeq and differential expression was run between aged HSC and young HSC with donor sex as a covariate. Young HSC and aged HSC-specific genes with log_2_FoldChange > 1 and FDR < 0.01 were retained as signatures for downstream analysis. For the Ainciburu dataset, DESeq was run on raw counts from young and aged HSC pseudobulks only within samples profiled by 10x 3’ scRNA-seq V2 chemistry to avoid technology-driven batch effects. This comparison in the Ainciburu et al. dataset was confounded by donor sex, wherein all aged samples were male, and all young samples were female. To attenuate this, sex specific genes (X-inactivation genes XIST and TSIX, as well as ChrY genes outside of the para-autologous region) were filtered out from the DE results. Young HSC and aged HSC-specific genes with log_2_FoldChange > 1 and FDR < 0.01 were retained as signatures for downstream analysis. For the Aksöz et al. dataset, raw count pseudobulks were modeled with EdgeR as implemented in the Libra (v1.0.0) package,^124^ and differential expression was run using a likelihood ratio test between aged HSC and young HSC. Gene identifiers were converted to GENCODE v38 and young HSC and aged HSC-specific genes with log_2_FoldChange > 1 and FDR < 0.01 were retained as signatures for downstream analysis, excluding genes not in the GENCODE reference annotation. Differential expression results are listed in Table S6.

The quality of each resulting signature was evaluated by scoring across donors within our CH cohort and evaluating their association with age. While we validated that aged HSC signatures from each dataset were positively correlated with age, young HSC signatures were uncorrelated with age, rather than having the expected negative correlation. Thus, only aged HSC signatures were used for downstream analysis (Table S5).

### Quantification and statistical analysis

Data analysis and statistical tests were performed using R version 4.2.1. Plots were generated using ggplot2 (v3.3.6) or FlowJo (v10.8.1). Detail on statistical tests used in the different figures and definition of relevant summary statistics are included in each figure legend.

## Supplemental information

Document S1. Figures S1–S6 and supplemental references

Table S1. Clinical characteristics of the MARCH cohort, related to Figure 1

Table S2. Targeted DNA sequencing, related to Figure 1

Table S3. Samples analyzed by TARGET-seq+, related to Figures 2 and 3

Table S4. Reaction conditions and primers used during TARGET-seq+ RT-PCR and barcoding PCR steps, custom i5 indexes for 3′ TARGET-seq+ transcriptome library amplification, and primer/probe sequences for ddPCR, related to Figures 2 and 3

Table S5. Hematopoietic stem/progenitor and lineage genesets used for GSEA and AUCell analyses, related to Figures 4, 5, and 6

Table S6. Differential expression results, related to Figures 4, 5, and 6

Document S2. Article plus supplemental information
