# Supplementary material for: Selective advantage of mutant stem cells in human clonal hematopoiesis is associated with attenuated response to inflammation and aging
Source: Cell Stem Cell. 2024 Aug 1;31(8):1127–1144.e17. doi: 10.1016/j.stem.2024.05.010 (PMC11512683; doi:10.1016/j.stem.2024.05.010)
Supplement: Document S1. Figures S1–S6 and supplemental references [file mmc1.pdf]

Cell Stem Cell, Volume *31*

Supplemental Information

Selective advantage of mutant stem cells in human clonal hematopoiesis is associated with attenuated response to inflammation and aging

Niels Asger Jakobsen, Sven Turkalj, Andy G.X. Zeng, Bilyana Stoilova, Marlen Metzner, Susann Rahmig, Murtaza S. Nagree, Sayyam Shah, Rachel Moore, Batchimeg Usukhbayar, Mirian Angulo Salazar, Grigore-Aristide Gafencu, Alison Kennedy, Simon Newman, Benjamin J.L. Kendrick, Adrian H. Taylor, Rasheed Afinowi-Luitz, Roger Gundle, Bridget Watkins, Kim Wheway, Debra Beazley, Alex Murison, Alicia G. Aguilar-Navarro, Eugenia Flores-Figueroa, Stephanie G. Dakin, Andrew J. Carr, Claus Nerlov, John E. Dick, Stephanie Z. Xie, and Paresh Vyas

SUPPLEMENTAL FIGURES AND LEGENDS

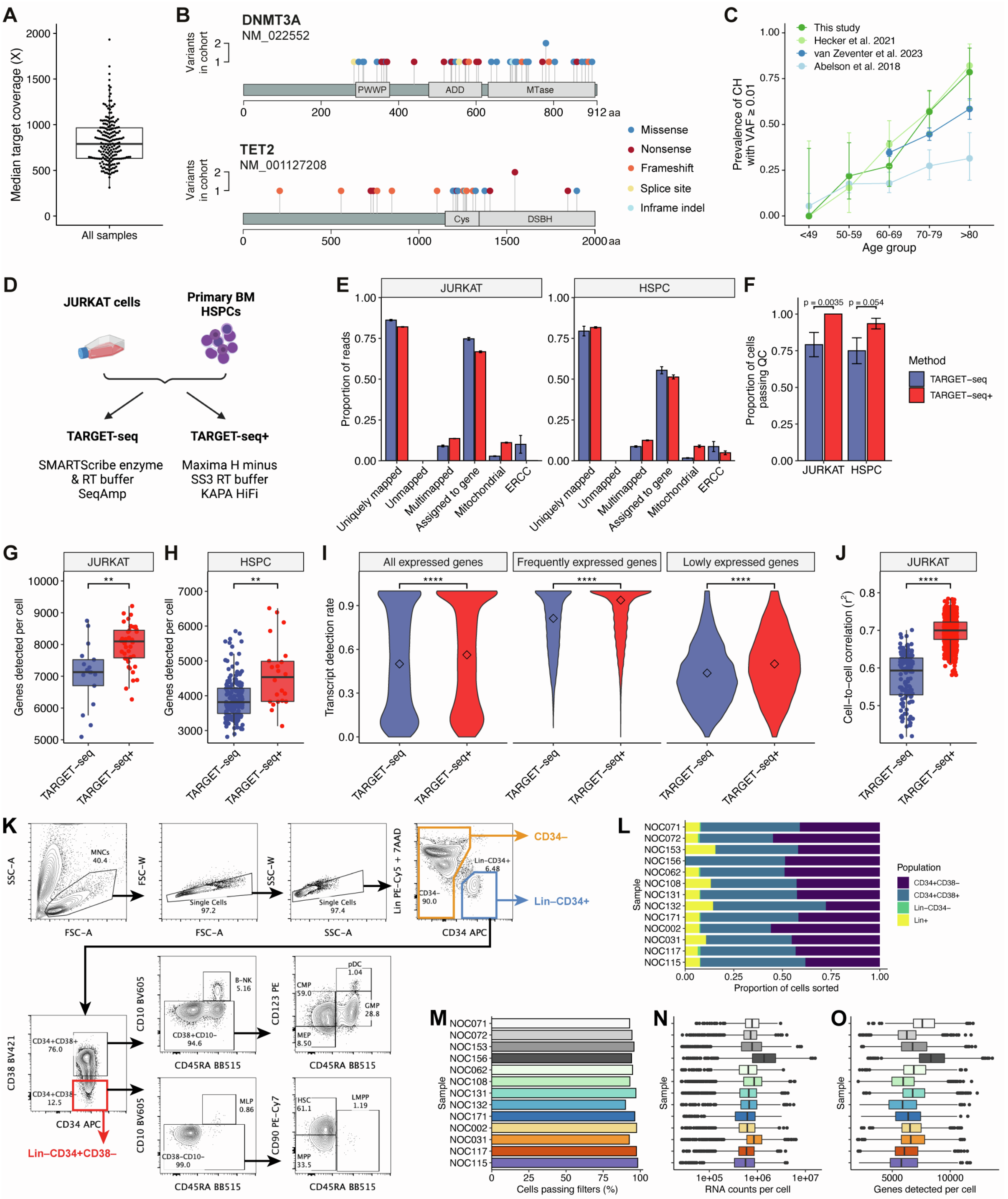

Figure S1. Identification of cases with age-related CH in individuals undergoing hip replacement surgery, validation of TARGET-seq+, and application of TARGET-seq+ to CH and WT samples, related to Figures 1 and 2 and STAR Methods.

(A) Median coverage across the target region (97 genes in total) in targeted DNA sequencing of BM samples. Each point represents a sample. The boxplot shows the median and interquartile range.

(B) Distribution of mutations in coding regions of *DNMT3A* and *TET2*. Positions of protein domains are shown: PWWP, proline-tryptophan-tryptophan-proline; ADD, ATRX-DNMT3-DNMT3L; MTase, cytosine methyltransferase; Cys, cysteine-rich; DSBH, double-stranded β-helix. Mutations are colored by the predicted effect on the protein.
(C) Prevalence of CH with at least one driver mutation (VAF ≥ 0.01) by age. BM DNA sequencing data from participants in this study (n = 195) are compared with another hip replacement cohort (Hecker et al.[1]; n = 109 BM and n = 91 PB samples), and with two other studies of the general population (Abelson et al.[2]; n = 676 PB samples, and van Zeventer et al.[3]; n = 3,359 PB samples). Error bars represent 95% confidence intervals. Studies performed on individuals undergoing hip replacement surgery are indicated in green, while other studies are indicated in blue colors.
(D) Experimental scheme for validation of TARGET-seq+ in JURKAT cells and primary human $CD34^+$ HSPCs. Metrics were compared to single cell transcriptome libraries generated with the original TARGET-seq protocol.
(E) Sequencing statistics of single cell transcriptome libraries from JURKAT and primary $CD34^+$ HSPCs processed with TARGET-seq and TARGET-seq+. Data are represented as the proportion ± SE.
(F) Proportion of cells passing quality control (QC) for transcriptome libraries, for each method (n = 24 JURKAT for TARGET-seq; 46 JURKAT for TARGET-seq+; 24 HSPC for TARGET-seq; and 46 HSPC for TARGET-seq+). Data are represented as the proportion ± SE. P-values calculated by Fisher's exact test.
(G and H) Number of genes detected per cell in JURKAT cells (G) and primary $CD34^+$ HSPCs (H). In both cases, reads were downsampled to 5×10^5 reads per cell. Each dot represents a cell, and each boxplot represents the median and first and third quartiles. P-values calculated by unpaired two-tailed *t*–test. **p < 0.01.
(I) Comparison of transcript detection rates between TARGET-seq and TARGET-seq+ for JURKAT cells. A subsample of 16 cells per chemistry was analyzed. Reads were downsampled to 5×10^5 reads per cell. Dropout frequencies are shown for all genes expressed in at least 2 cells (left), for genes expressed in > 50% of cells by any method (middle), and for genes with low expression (mean normalized counts of 2-10 per cell; right). Diamonds show the median detection rate for each condition. P-values calculated by two-tailed Wilcoxon rank-sum test.
(J) Quantification of the reproducibility of gene expression in JURKAT cells, for TARGET-seq (n = 17 cells) and TARGET-seq+ (n = 43 cells). r^2 values for all pairwise cell-to-cell Pearson's correlations in libraries downsampled to 5×10^5 reads per cell are shown. P-values calculated by unpaired two-tailed *t*–test. ****p < 0.0001.

(K) FACS index sorting strategy for TARGET-seq+ experiments. Sorting gates for $Lin^{-}CD34^{+}$ (blue), $Lin^{-}CD34^{+}CD38^{-}$ (red), and $CD34^{-}$ (orange) cells are shown.

(L) Proportion of cells obtained in each immunophenotype for TARGET-seq+ analysis in each sample.

Panels (M) to (O) show TARGET-seq+ transcriptome quality control and sequencing statistics by sample. In panels (N) and (O), boxplots show the median and interquartile range.

(M) Percentage of cells passing quality control filters.

(N) Sequencing depth (RNA counts) per cell.

(O) Number of genes detected per cell.

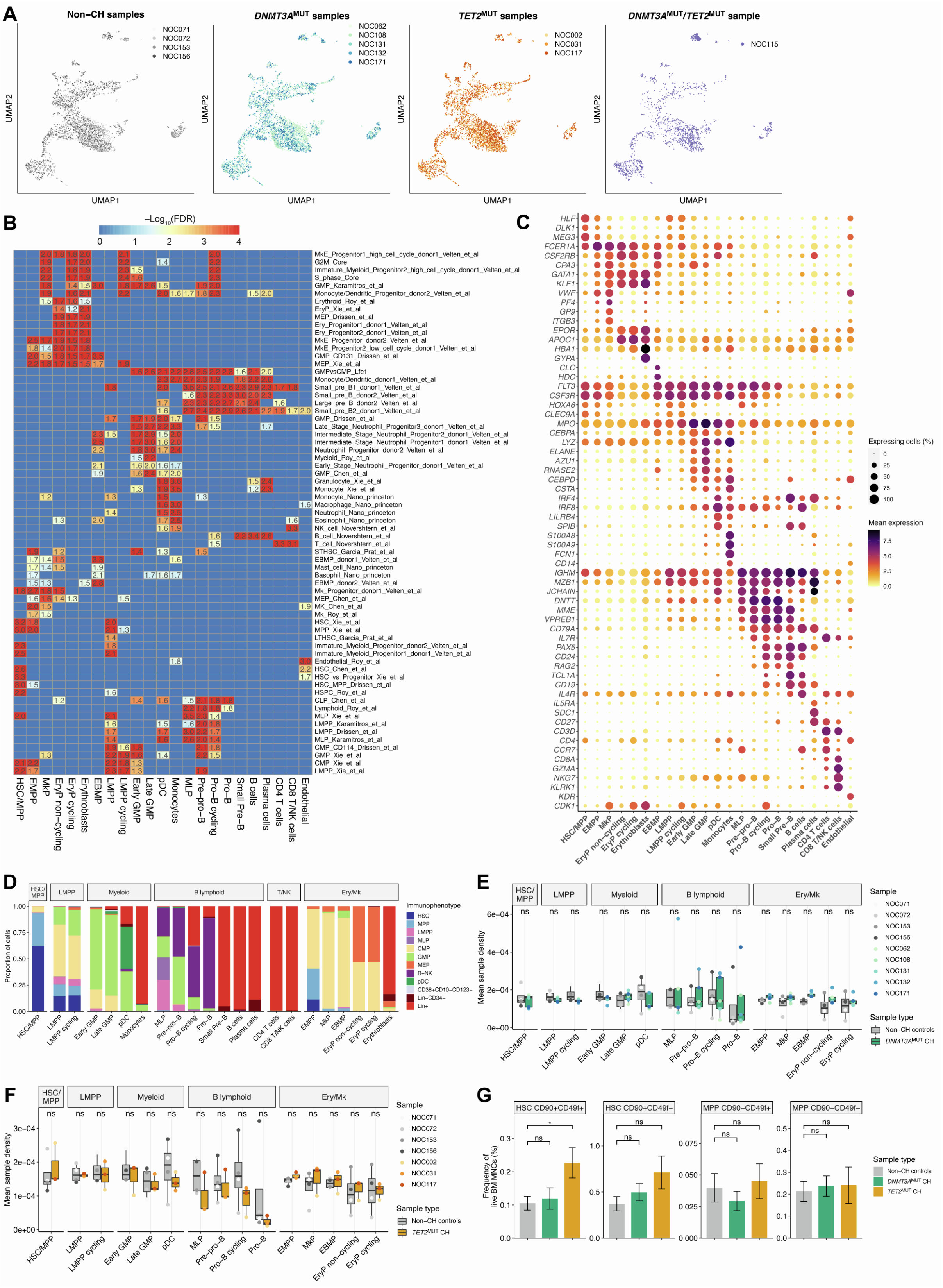

Figure S2. TARGET-seq+ applied to *DNMT3A*- and *TET2*-mutant clonal hematopoiesis, related to Figure 2 and STAR Methods.

(A) UMAPs colored by sample, split by sample genotype. Colors indicate cells from different samples.
(B) Heatmap of GSEA results for genes differentially expressed between each cluster and all other clusters, using published gene signatures for HSPCs.[4-12] Colors represent $-\log_{10}$ of the false discovery rate (FDR). Numbers inside the heatmap indicate the normalized enrichment score for each comparison, showing only those with FDR < 0.05.
(C) Expression of marker genes by cluster. The size of each dot represents the percentage of cells expressing each gene and the color represents the mean expression level (log normalized counts).
(D) Proportions of each immunophenotypic population in each transcriptional cluster (x-axis).
(E) Boxplots summarizing Figure 2F, showing the mean density of cells per cluster in *DNMT3A*^{MUT} CH samples (green) compared to WT control samples (grey). Median and interquartile range are shown. Each dot represents the mean value from a single sample (color legend on the right). No credible differences between *DNMT3A*^{MUT} CH samples and control samples were identified (with FDR < 0.2) using scCODA.
(F) As in (E) summarizing Figure 2G, comparing *TET2*^{MUT} CH samples (orange) with WT control samples (grey). No credible differences between *TET2*^{MUT} CH samples and control samples were identified (with FDR < 0.2) using scCODA.
(G) Barplots showing the frequency of CD90$^{+/-}$ and CD49f$^{+/-}$ HSC/MPP cells as a percentage of total BM MNCs. Data are represented as mean ± SEM. P-values calculated by Wilcoxon rank-sum test with Holm-Bonferroni multiple testing correction. *p < 0.05.
HSC, hematopoietic stem cell; MPP, multipotent progenitor; EMPP, erythroid/megakaryocyte-primed multipotent progenitor; MkP, megakaryocytic progenitor; EryP, erythroid progenitor; EBMP, Eosinophil-basophil-mast cell progenitor; LMPP, lymphoid-primed multipotent progenitor; GMP, granulocyte-monocyte progenitor; pDC, plasmacytoid dendritic cell progenitor; MLP, multi-lymphoid progenitor; B-NK, B and NK cell progenitor.

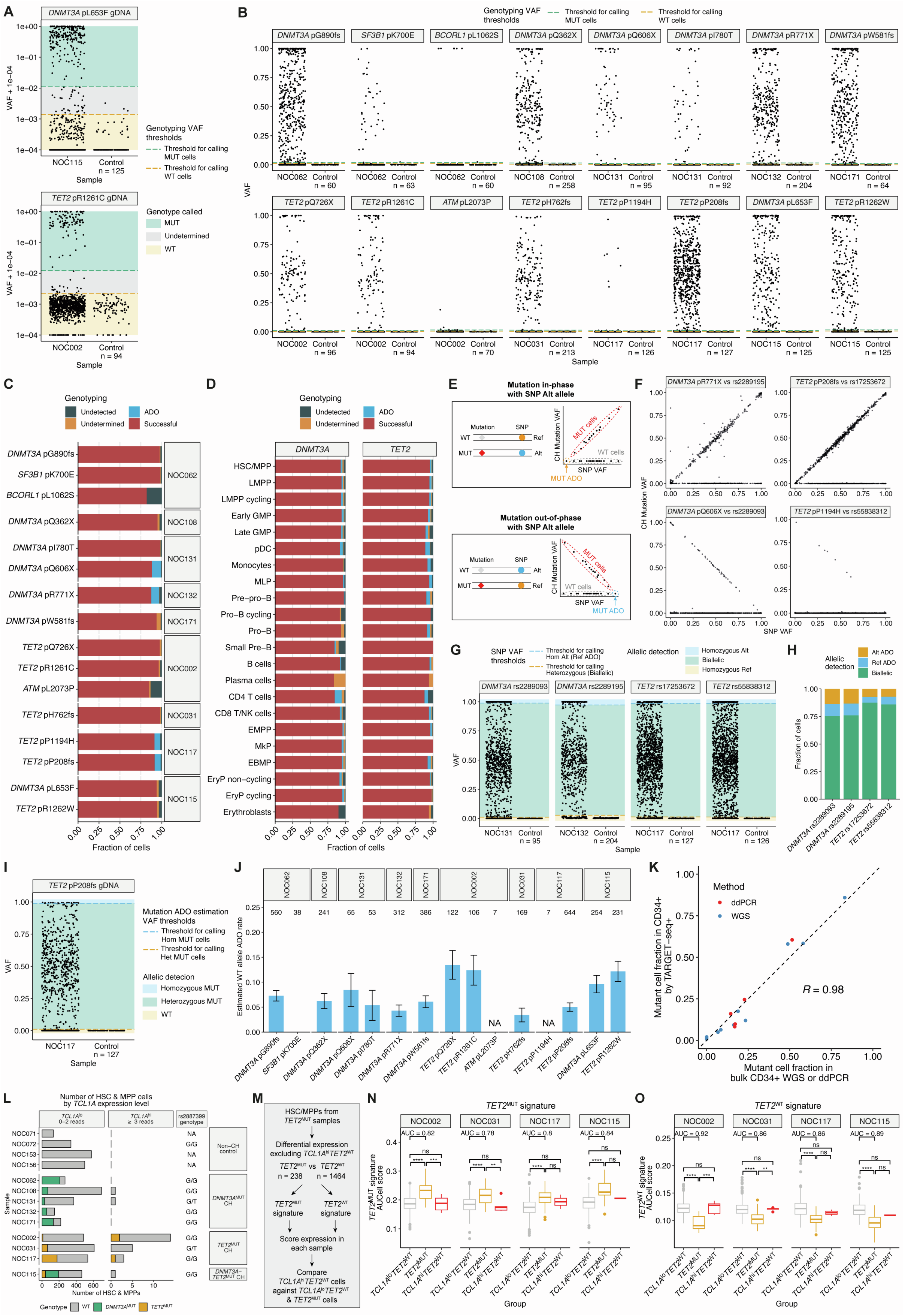

Figure S3. TARGET-seq+ single-cell genotyping quality control and validation, related to Figure 3 and STAR Methods.

(A) Single-cell variant allele frequency (scVAF) distribution (y-axis; logarithmic scale) obtained from gDNA amplicons for two representative mutations in *DNMT3A* and *TET2*, comparing CH samples (on the left of each graph) versus the WT control sample (on the right of each graph). Each dot represents a single cell. Cells below the lower threshold (orange) are called WT; cells between the two thresholds (grey) are called undetermined; cells above the upper threshold (green) are called mutant (detailed in STAR Methods).

(B) Plots of scVAF distribution (y-axis, linear scale) from gDNA amplicons across all mutant loci, comparing CH samples (on the left of each graph) versus WT control samples (on the right of each graph). Each dot represents a single cell. Thresholds for calling cells mutant or WT are shown.

(C) Fraction of cells successfully genotyped (red) for each mutation and sample analyzed. In 4 amplicons, allelic dropout (ADO) of the mutant allele (blue) could be identified by analysis of a germline heterozygous SNP in the same amplicon (STAR Methods). Cells with borderline VAF were assigned an undetermined genotype (orange) for that mutation.

(D) Fraction of cells successfully genotyped for *DNMT3A* and *TET2* across transcriptionally defined cell types.

(E) Schematic overview of how germline heterozygous SNPs may be phased with mutations in the same genotyping amplicon. When the SNP Alternate (Alt) allele is in-phase with the mutation (i.e. they are located on the same allele, top), cells where there has been ADO of the mutant allele will have a single-cell variant allele frequency (scVAF) close to 0% for both the mutation and the SNP. When the SNP Alt allele is out-of-phase with the mutation (i.e. they are located on opposite alleles, bottom), cells where there has been ADO of the mutant allele will have a scVAF of ~0% for the mutation but a scVAF of ~100% for the SNP.

(F) scVAF of mutations (y-axis) plotted against the scVAF of germline heterozygous SNPs (x-axis) found in the same amplicon. Each dot represents a single cell. The SNP and the mutation are either in-phase, i.e. they are located on the same allele (top plots), or they are out-of-phase, i.e. they are located on opposite alleles (bottom plots), as evidenced by the correlation in scVAF in mutant cells.

(G) Plots of scVAF distribution (y-axis; linear scale) across 4 germline heterozygous SNPs used for ADO detection in gDNA amplicons (STAR Methods). Each dot represents a single cell. Cells from the CH samples are plotted on the left and cells from the WT control sample are plotted on the right of each graph. The WT control sample was homozygous reference for all 4 SNPs. Cells below the lower threshold (orange) are called Homozygous Ref; cells between the two thresholds (green) are called heterozygous (Biallelic detection); cells above the upper threshold (blue) are called Homozygous Alt.

(H) Frequency of biallelic and mono-allelic detection of 4 germline heterozygous SNPs co-amplified with somatic mutations.

(I) Plot of scVAF distribution as in (B), but for a representative *TET2* mutation, showing how homozygous mutant cells were called. Cells above the lower threshold (orange) are called mutant (MUT); cells above the upper threshold (blue) are called Homozygous MUT. The threshold for calling cells homozygous mutant is the inverse of the lower threshold. The frequency of cells called Homozygous MUT was used to estimate ADO rates (see panel J).

(J) Bar plots showing the estimated WT ADO rate for each mutant locus. The hemizygous *BCORL1* locus was excluded. Samples are indicated on the top. Numbers above each bar show the number of mutant cells analyzed for each locus. Error bars indicate the standard error. NA indicates those mutations for which the ADO rate was not calculated due to the small number of mutant cells.

(K) Comparison of the mutant cell fraction for each mutation identified in Lin^-CD34^+ HSPCs by TARGET-seq+ (y-axis) with the clonal cell fraction in bulk Lin^-CD34^+ HSPCs (x-axis) as measured by whole genome sequencing or ddPCR. *R* indicates the Pearson correlation coefficient.

(L) Number of immunophenotypic HSC & MPP cells in non-CH and CH samples according to *TCL1A* expression level. Bars are colored by cell genotype. Where available, the rs2887399 genotype is shown for each sample. NA, not available.

(M) Strategy for analysis of genotype-specific expression profile in $TCL1A^{hi}TET2^{WT}$ HSC/MPP. $TET2^{MUT}$ and $TET2^{WT}$ signatures were defined based on differential expression analysis between $TET2^{MUT}$ and $TET2^{WT}$ HSC/MPPs from the $TET2^{MUT}$ CH samples (excluding $TCL1A^{hi}TET2^{WT}$ cells).

(N-O) AUCell scores for the $TET2^{MUT}$ (N) and $TET2^{WT}$ (O) signatures in immunophenotypic HSC/MPPs from $TET2^{MUT}$ CH samples. Two populations of $TET2^{WT}$ cells are shown: $TCL1A^{hi}TET2^{WT}$ cells (defined as WT cells with > 2 *TCL1A* reads) and $TCL1A^{lo}TET2^{WT}$ (defined as WT cells with ≤ 2 *TCL1A* reads). Performance of each signature in predicting $TET2^{MUT}$ vs $TET2^{WT}$ genotype is shown by the area under the ROC curve (AUC). P-values calculated by two-tailed Wilcoxon rank-sum test. **p < 0.01, ***p < 0.001, ****p < 0.0001.

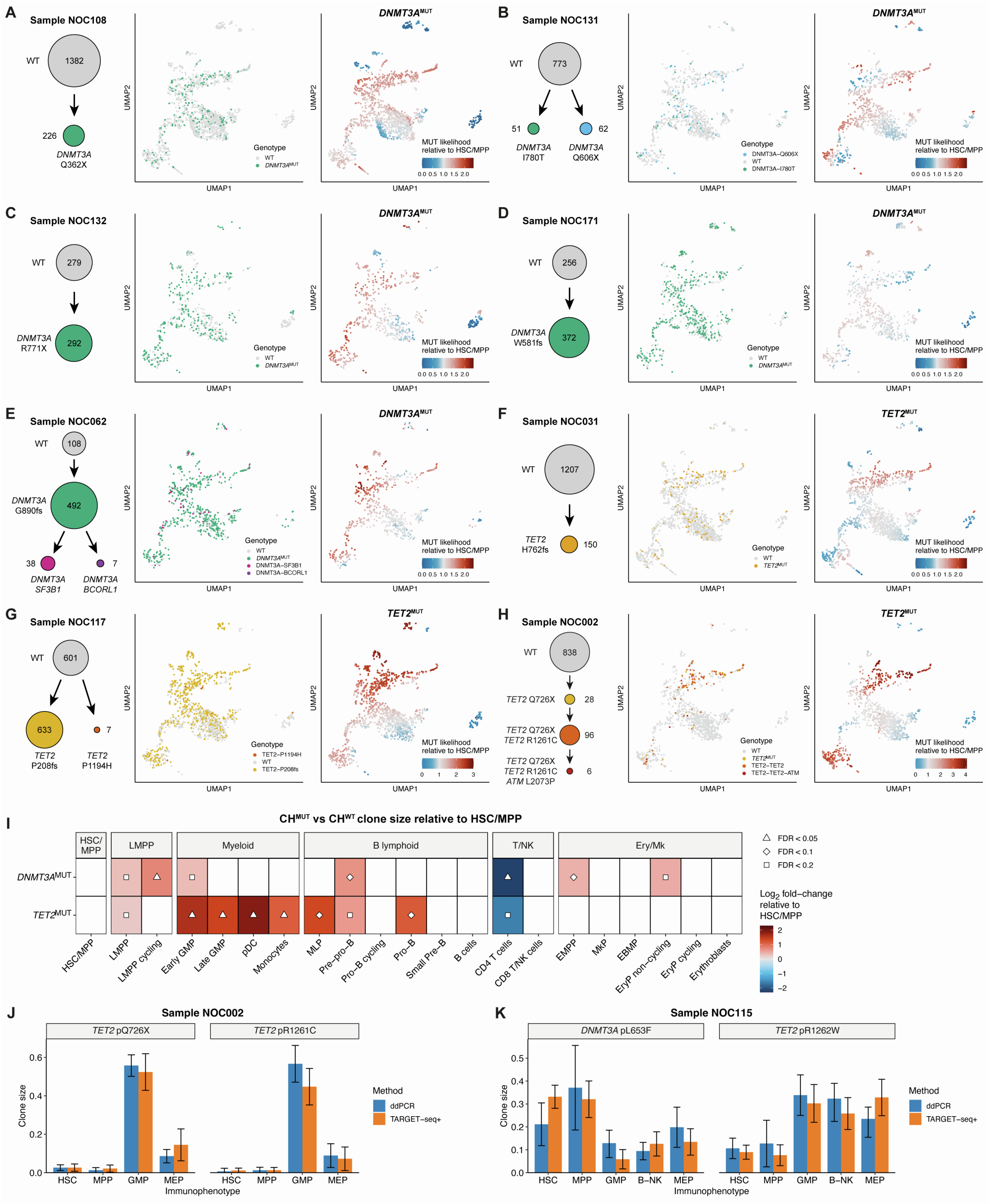

Figure S4. Sample-specific patterns of clonal expansion of *DNMT3A*- and *TET2*-mutant clones, related to Figure 3.

(A) to (H) Clonal structures and patterns of clonal expansion for individual *DNMT3A*- and *TET2*-mutant CH samples. For each sample, the clonal structure determined by single-cell genotyping is shown on the left. Numbers of cells assigned to each genotype are shown. The middle panels show successfully genotyped cells from each sample on the UMAP embedding, labelled by cell genotype. Right-hand panels show the relative likelihood for mutant clones

carrying a single mutation in *DNMT3A* or *TET2* on the UMAP. In each case, the mutant clone likelihood is normalized to the mean likelihood in the HSC/MPP cluster. Values > 1 indicate clonal expansion relative to HSC/MPP and values < 1 indicate smaller clone size relative to HSC/MPP.

(I) Heatmap of log_2FC in abundance of mutant clones relative to the HSC/MPP as estimated with scCODA. Only significant results at an FDR < 0.2 are shown, with all nonsignificant differences plotted as white. Symbols (legend on the right) indicate whether results are significant at FDR threshold of 0.05, 0.1 or 0.2.

(J) Mutant clone size for two *TET2* mutations in sample NOC002 within immunophenotypic populations. Data from ddPCR of FACS-sorted populations (blue bars) is compared with frequencies of mutant cells in TARGET-seq+ data (orange bars). Error bars represent 95% confidence intervals.

(K) Same as in (J) but for the *DNMT3A* and *TET2* mutations in sample NOC115.

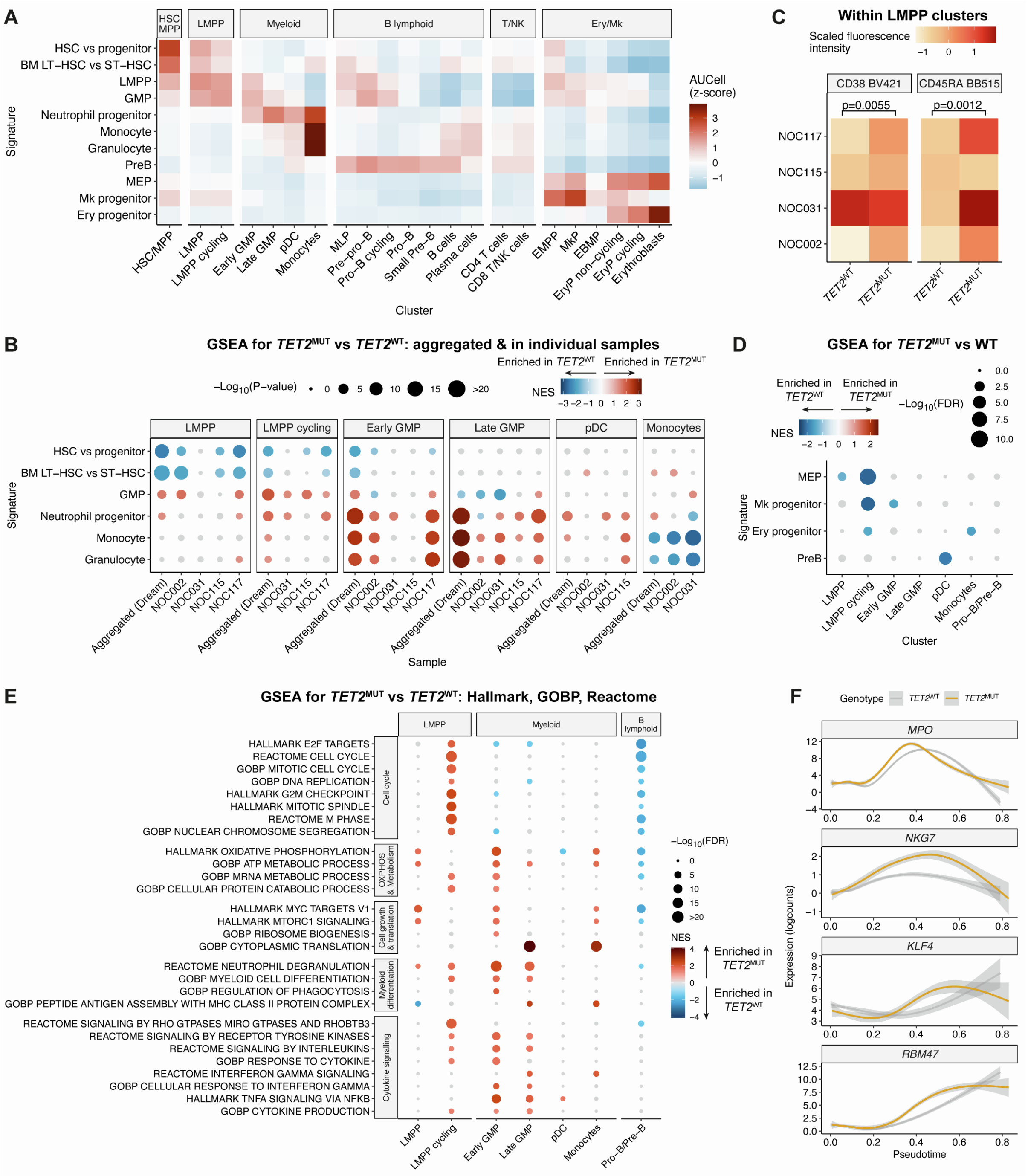

Figure S5. Consequences of *TET2* mutations in myeloid progenitors, related to Figure 4.

(A) Heatmap of scaled AUCell expression scores for HSC, myeloid, B-lymphoid, megakaryocytic, and erythroid gene signatures (y-axis) used in Figures 4C, 4D, S5B and S5D, by cluster (x-axis). See also Table S5.

(B) Comparison of GSEA results between the aggregated Dream linear mixed model and differential expression analysis in individual samples using a Wilcox test. GSEA was performed against hematopoietic signatures comparing *TET2*^{MUT} versus *TET2*^{WT} cells within each LMPP and GMP cluster. Results are not shown for samples with < 3 cells in either

genotype in a particular cluster. Color intensity indicates the normalized enrichment score (NES); positive NES values indicate enrichment in mutant cells. Signatures with P-value > 0.05 are colored grey.

(C) Expression of CD38 and CD45RA cell surface protein, measured by FACS indexing in *TET2*^{WT} and *TET2*^{MUT} cells, in the LMPP clusters. P-values calculated by linear mixed model.

(D) GSEA against erythroid, megakaryocytic, and lymphoid gene signatures, comparing *TET2*^{MUT} against *TET2*^{WT} cells within each lympho-myeloid cluster. Pre-pro-B, Pro-B, and Small Pre-B cells were combined for the Pro-B/Pre-B analysis. Differential expression analysis was performed accounting for sample and batch effects. Cells from the 4 *TET2*^{MUT} CH samples were included. Signatures with FDR > 0.05 are colored grey. Positive NES values indicate enrichment in mutant cells.

(E) GSEA against Hallmark, Gene Ontology biological process (GOBP), and Reactome signatures comparing *TET2*^{MUT} against *TET2*^{WT} cells within the LMPP, GMP, pDC, monocyte, and Pro-B/Pre-B clusters. Signatures with FDR > 0.05 are colored grey. Positive NES values indicate enrichment in mutant cells.

(F) Local regression of gene expression values along myeloid pseudotime for myeloid lineage-affiliated genes, comparing *TET2*^{MUT} and *TET2*^{WT} cells.

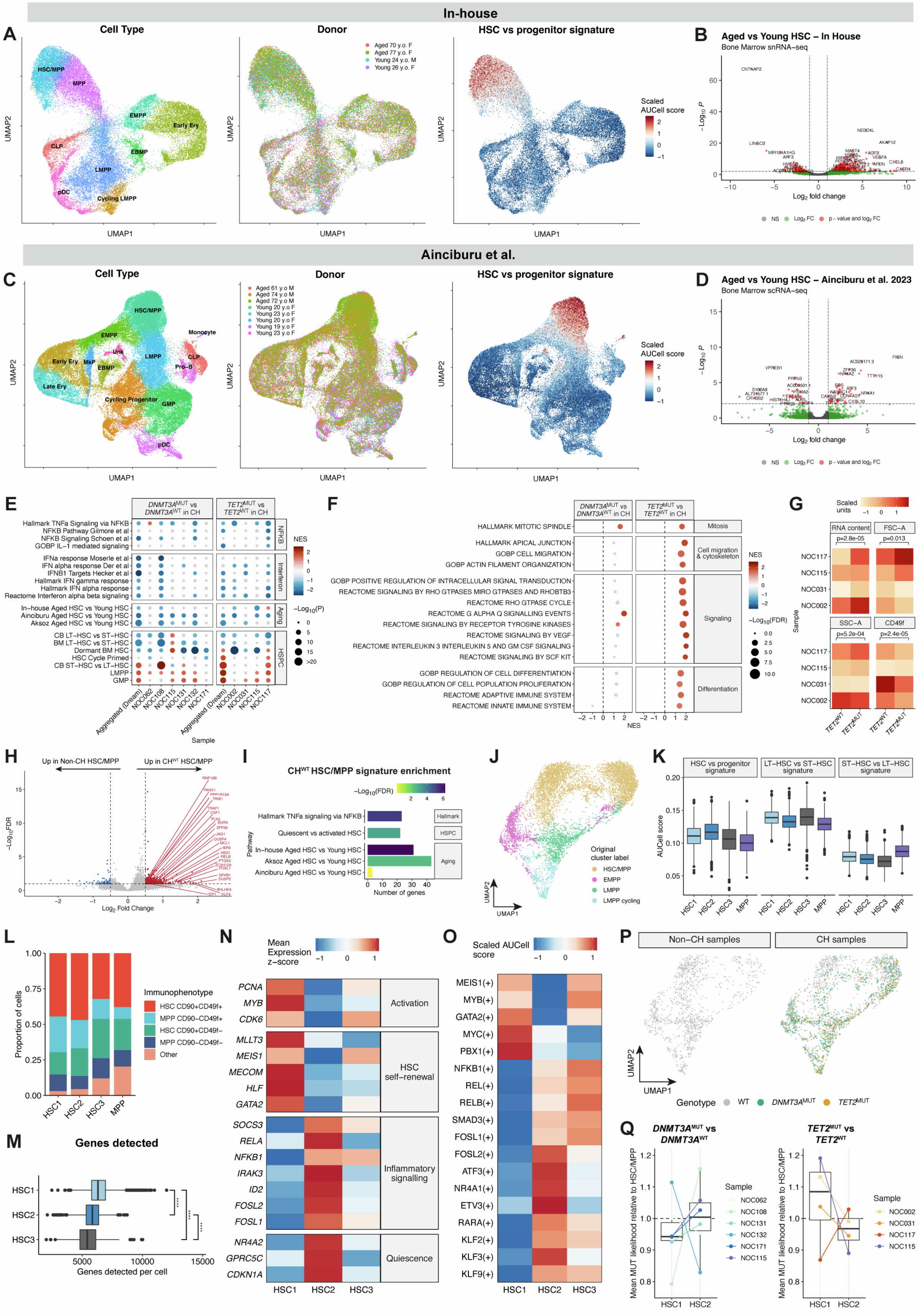

Figure S6. Generation of aged HSC gene signatures, consequences of CH mutations in

HSC/MPP, and characterization of HSC transcriptional heterogeneity, related to Figures 5 and 6.

(A) UMAPs showing snRNA-seq data for BM HSPCs from 2 young and 2 aged donors (in-house dataset) colored by Louvain cluster identity (left), donor identity (middle) and the scaled AUCell score for the HSC vs progenitor signature (right).

(B) Volcano plot showing differentially expressed genes (DEGs) between HSC/MPP from aged donors versus those from young donors from the in-house dataset. Genes with FDR < 0.01 and $\log_2$FC > 1 were considered differentially expressed (labelled red).

(C) UMAPs showing snRNA-seq data for BM HSPCs from 5 young and 3 aged donors (Ainciburu et al. dataset[13]) colored by Louvain cluster identity (left), donor identity (middle), and the scaled AUCell score for the HSC vs progenitor signature (right).

(D) Volcano plot showing DEGs between HSC/MPP from aged donors versus those from young donors from the Ainciburu et al[13] dataset. Genes with FDR < 0.01 and $\log_2$FC > 1 were considered differentially expressed (labelled red).

(E) Comparison of GSEA results between the aggregated Dream linear mixed model and differential expression analysis in individual samples using a Wilcox test. GSEA against NF-κB, interferon, and hematopoietic signatures comparing *DNMT3A*^{MUT} versus *DNMT3A*^{WT} HSC/MPPs and *TET2*^{MUT} versus *TET2*^{WT} HSC/MPPs within CH samples. Signatures with P-value > 0.05 are colored grey. Positive NES values indicate enrichment in mutant cells.

(F) GSEA against Hallmark, Gene Ontology biological process (GOBP), and Reactome signatures, comparing *DNMT3A*^{MUT} against *DNMT3A*^{WT} HSC/MPPs (left) and *TET2*^{MUT} against *TET2*^{WT} HSC/MPPs (right), within CH samples. Signatures with FDR > 0.2 are colored grey. Positive NES values indicate enrichment in mutant cells.

(G) RNA content (ratio between endogenous RNA reads and ERCC spike-in reads), cell size, granularity, and CD49f protein expression measured by FACS indexing, comparing *TET2*^{WT} against *TET2*^{MUT} HSC/MPPs. P-values calculated by linear mixed model.

(H) Volcano plot showing DEGs between CH^{WT} HSC/MPPs from 9 CH samples (n = 2622 cells) and WT HSC/MPPs from 4 non-CH samples (n = 1279 cells). Genes with FDR < 0.1 and $\log_2$FC > 0.5 were considered differentially expressed. Genes in the TNFα via NFKB pathway are labelled in red.

(I) Pathway enrichment of genes upregulated in CH^{WT} versus non-CH HSC/MPPs against Hallmark and hematopoietic signatures. Significance calculated by hypergeometric test.

(J) UMAP embedding of 8059 cells from the HSC/MPP, EMPP, LMPP and LMPP cycling clusters after feature weight derivation with the Self-Assembling Manifolds (SAM) algorithm. Cells are colored by the original cluster annotation used in previous figures.

(K) Comparison of AUCell expression scores for HSC gene signatures (indicated above each graph) in HSC and MPP subclusters (x-axis).

(L) Immunophenotype of cells in HSC subclusters (x-axis).

(M) Boxplot showing the number of genes detected per cell within the HSC1-3 clusters. ****p < 0.0001. Boxplots display the median and interquartile range. P-values calculated by unpaired *t*-test.

(N) Expression of genes related to HSC quiescence, activation, self-renewal, and inflammatory signaling within the HSC1-3 clusters.

(O) Heatmap showing scaled AUCell scores for selected regulons that are differentially active in the HSC1-3 clusters.

(P) UMAP embeddings showing cells from non-CH and CH samples. Cells are colored by genotype. For clarity, only WT and single-mutant cells are shown.

(Q) Boxplots showing the distribution of mean mutant clone likelihood in the HSC1 and HSC2 clusters, within *DNMT3A*^{MUT} and *TET2*^{MUT} CH samples. Boxplots display the median and interquartile range. Each dot represents a CH sample (color legend on the right). Mutant clone likelihood is normalized to the mean within the HSC/MPP clusters.

Supplemental References

1. Hecker, J.S., Hartmann, L., Rivière, J., Buck, M.C., van der Garde, M., Rothenberg-Thurley, M., Fischer, L., Winter, S., Ksienzyk, B., Ziemann, F., et al. (2021). CHIP and hips: clonal hematopoiesis is common in patients undergoing hip arthroplasty and is associated with autoimmune disease. Blood *138*, 1727-1732. https://doi.org/10.1182/blood.2020010163.

2. Abelson, S., Collord, G., Ng, S.W.K., Weissbrod, O., Mendelson Cohen, N., Niemeyer, E., Barda, N., Zuzarte, P.C., Heisler, L., Sundaravadanam, Y., et al. (2018). Prediction of acute myeloid leukaemia risk in healthy individuals. Nature *559*, 400-404. https://doi.org/10.1038/s41586-018-0317-6.

3. van Zeventer, I.A., de Graaf, A.O., Salzbrunn, J.B., Nolte, I.M., Kamphuis, P., Dinmohamed, A., van der Reijden, B.A., Schuringa, J.J., Jansen, J.H., and Huls, G. (2023). Evolutionary landscape of clonal hematopoiesis in 3,359 individuals from the general population. Cancer Cell. https://doi.org/10.1016/j.ccell.2023.04.006.

4. Velten, L., Haas, S.F., Raffel, S., Blaszkiewicz, S., Islam, S., Hennig, B.P., Hirche, C., Lutz, C., Buss, E.C., Nowak, D., et al. (2017). Human haematopoietic stem cell lineage commitment is a continuous process. Nature Cell Biology *19*, 271-281. https://doi.org/10.1038/ncb3493.

5. Novershtern, N., Subramanian, A., Lawton, L.N., Mak, R.H., Haining, W.N., McConkey, M.E., Habib, N., Yosef, N., Chang, C.Y., Shay, T., et al. (2011). Densely Interconnected Transcriptional Circuits Control Cell States in Human Hematopoiesis. Cell *144*, 296-309. https://doi.org/10.1016/j.cell.2011.01.004.

6. Drissen, R., Thongjuea, S., Theilgaard-Mönch, K., and Nerlov, C. (2019). Identification of two distinct pathways of human myelopoiesis. Science Immunology *4*, eaau7148-eaau7148. https://doi.org/10.1126/sciimmunol.aau7148.

7. Roy, A., Wang, G., Iskander, D., O'Byrne, S., Elliott, N., O'Sullivan, J., Buck, G., Heuston, E.F., Wen, W.X., Meira, A.R., et al. (2021). Transitions in lineage specification and gene regulatory networks in hematopoietic stem/progenitor cells over human development. Cell Reports *36*, 109698-109698. https://doi.org/10.1016/j.celrep.2021.109698.

8. Xie, S.Z., Kaufmann, K.B., Wang, W., Chan-Seng-Yue, M., Gan, O.I., Laurenti, E., Garcia-Prat, L., Takayanagi, S.-i., Ng, S.W.K., Xu, C., et al. (2021). Sphingosine-1-Phosphate Receptor 3 Potentiates Inflammatory Programs in Normal and Leukemia Stem Cells to Promote Differentiation. Blood Cancer Discovery *2*, 32-53. https://doi.org/10.1158/2643-3230.BCD-20-0155.

9. Karamitros, D., Stoilova, B., Aboukhalil, Z., Hamey, F., Reinisch, A., Samitsch, M., Quek, L., Otto, G., Repapi, E., Doondeea, J., et al. (2018). Single-cell analysis reveals the continuum of human lympho-myeloid progenitor cells. Nature Immunology *19*, 85-97. https://doi.org/10.1038/s41590-017-0001-2.

10. Chen, L., Kostadima, M., Martens, J.H.A., Canu, G., Garcia, S.P., Turro, E., Downes, K., Macaulay, I.C., Bielczyk-Maczynska, E., Coe, S., et al. (2014). Transcriptional diversity during lineage commitment of human blood progenitors. Science *345*, 1251033-1251033. https://doi.org/10.1126/science.1251033.

11. Tirosh, I., Izar, B., Prakadan, S.M., Wadsworth, M.H., Treacy, D., Trombetta, J.J., Rotem, A., Rodman, C., Lian, C., Murphy, G., et al. (2016). Dissecting the multicellular ecosystem of metastatic melanoma by single-cell RNA-seq. Science *352*, 189-196. https://doi.org/10.1126/science.aad0501.

12. Zhang, Y.W., Mess, J., Aizarani, N., Mishra, P., Johnson, C., Romero-Mulero, M.C., Rettkowski, J., Schönberger, K., Obier, N., Jäcklein, K., et al. (2022). Hyaluronic acid–GPRC5C signalling promotes dormancy in haematopoietic stem cells. Nature Cell Biology *24*, 1038-1048. https://doi.org/10.1038/s41556-022-00931-x.

13. Ainciburu, M., Ezponda, T., Berastegui, N., Alfonso-Pierola, A., Vilas-Zornoza, A., San Martin-Uriz, P., Alignani, D., Lamo-Espinosa, J., San-Julian, M., Jiménez-Solas, T., et al. (2023). Uncovering perturbations in human hematopoiesis associated with healthy aging and myeloid malignancies at single-cell resolution. eLife *12*, e79363. https://doi.org/10.7554/eLife.79363.
